# Live-cell imaging reveals the relative contributions of antigen-presenting cell subsets to thymic central tolerance

**DOI:** 10.1038/s41467-019-09727-4

**Published:** 2019-05-17

**Authors:** J. N. Lancaster, H. M. Thyagarajan, J. Srinivasan, Y. Li, Z. Hu, L. I. R. Ehrlich

**Affiliations:** 1 Department of Molecular Biosciences, Institute of Cellular and Molecular Biology, The University of Texas at Austin, 100 E. 24th Street A5000, Austin, TX 78712 USA; 2 Livestrong Cancer Institutes, Dell Medical School, The University of Texas at Austin, Austin, TX 78712 USA

**Keywords:** Lymphocyte differentiation, Central tolerance, Antigen-presenting cells, Imaging the immune system

## Abstract

Both medullary thymic epithelial cells (mTEC) and dendritic cells (DC) present tissue-restricted antigens (TRA) to thymocytes to induce central tolerance, but the relative contributions of these antigen-presenting cell (APC) subsets remain unresolved. Here we developed a two-photon microscopy approach to observe thymocytes interacting with intact APCs presenting TRAs. We find that mTECs and DCs cooperate extensively to induce tolerance, with their relative contributions regulated by the cellular form of the TRA and the class of major histocompatibility complex (MHC) on which antigen is presented. Even when TRA expression is restricted to mTECs, DCs still present self-antigens at least as frequently as mTECs. Notably, the DC subset cDC2 efficiently acquires secreted mTEC-derived TRAs for cross-presentation on MHC-I. By directly imaging interactions between thymocytes and APCs, while monitoring intracellular signaling, this study reveals that distinct DC subsets and AIRE^+^ mTECs contribute substantially to presentation of diverse self-antigens for establishing central tolerance.

## Abstract (web summary)

Both thymic epithelial cells and dendritic cells present self antigens in the thymus to mediate thymic selection and T cell tolerance. Here the authors quantify, using two-photon live imaging of mouse thymic slices, the relative contribution of these two cell types, as well as the effects of antigen cross-presentation by dendritic cells, during tolerance induction.

## Introduction

The thymic medulla induces central tolerance to the myriad self-proteins that T cells encounter throughout the body^1^. When thymocytes recognize auto-antigens displayed by medullary antigen-presenting cells (APCs), they undergo either apoptosis, known as negative selection, or diversion to the regulatory T-cell (Treg) lineage. The two major thymic APC types that induce central tolerance are mature medullary thymic epithelial cells (mTECs) and dendritic cells (DCs). Though it is well-documented that thymocytes must access the medulla to establish central tolerance^2,3^, the relative contributions of mTECs versus DCs to tolerance induction remain unresolved^4^.

mTECs are well-suited to induce central tolerance against diverse self-antigens. Mature mTECs express about 90% of the proteome^5–7^, including tissue-restricted antigens (TRA), which are otherwise expressed by a limited number of tissues. AIRE, a transcriptional regulator that impacts mTEC maturation^8,9^, is required for expression of many TRAs^5,6,10^. The importance of AIRE^+^ mTECs to central tolerance is underscored by multi-organ autoimmunity in *Aire*^−/−^ mice or patients with *AIRE* deficiency^10,11^. In addition to expressing numerous self-antigens, mTECs express major histocompatibility complex class I (MHC-I), class II (MHC-II), and costimulatory molecules CD80 and CD86^12^, enabling them to directly present self-antigens to CD8^+^ and CD4^+^ single positive (CD8SP and CD4SP) thymocytes to induce tolerance^13–17^. However, any given TRA is expressed by only 1–3% of AIRE^+^ mTECs^18^, potentially limiting engagement with rare antigen-specific thymocytes.

Thymic DCs also express high levels of MHC-I, MHC-II, and costimulatory molecules^19^ and are thus poised to cooperate with mTECs to present self-antigens to thymocytes. DCs have been shown to acquire self-antigens from the blood^20,21^ and to traffic antigens into the thymus from peripheral tissues^22^. Elimination of DCs results in impaired central tolerance and autoimmunity, demonstrating their essential role in tolerance induction^23^. Notably, DCs can acquire and display TRAs expressed by mTECs, potentially distributing sparse self-antigens for a more efficient encounter by thymocytes^1, 24–26^.

To date, studies addressing the relative contributions of mTECs and DCs to central tolerance have used genetic models that either ablate mTECs or DCs, or inhibit their ability to present self-antigens^13–17,23, 27–31^. Although these studies address the intrinsic capacity of mTECs or DCs to mediate selection in the absence of the other cell type, and can identify TCRs via repertoire sequencing that require either APC subset for selection, they cannot address the roles of mTECs and DCs when both are intact. Importantly, crosstalk between thymocytes and stromal cells regulates differentiation and homeostasis of multiple thymic cell types: mTECs and DCs do not properly mature without signals from self-reactive CD4SP thymocytes^32–36^. Also, medullary localization and survival of some DC subsets depend on signals from mature mTECs^37–39^. Thus, genetically altering one stromal cell subset can impair maturation of others, making it difficult to deduce the physiologic contribution of different APCs to central tolerance.

To quantify the contributions of mTECs and DCs to central tolerance in a live thymic environment with intact APCs, we established a two-photon fluorescence microscopy (2PM) approach to directly visualize thymocyte:APC interactions within the thymic medulla, while simultaneously monitoring signaling driven by self-antigen recognition. A distinct advantage of this approach is its ability to reveal the redundant capacity of both mTECs and DCs to present a given TRA to induce tolerance of a monoclonal thymocyte population. Indeed, we find that both AIRE^+^ mTECs and DCs contribute substantially to presentation of a single TRA to CD4SP and CD8SP thymocytes, although the relative contribution of each APC varies according to the subcellular localization of the TRA and presentation on MHC-I versus MHC-II. TRAs expressed exclusively by AIRE^+^ mTECs are presented by DCs at least as efficiently as by AIRE^+^ mTECs themselves. Notably, Sirpα^+^ DCs (cDC2)^40^ are more efficient than Sirpα^−^ DCs (cDC1) at cross-presenting a secreted TRA expressed by AIRE^+^ mTECs. Consistent with data from monoclonal thymocytes, both AIRE^+^ mTECs and DCs efficiently present endogenous self-antigens to polyclonal CD4SP and CD8SP thymocytes, with a slightly greater contribution by DCs. These findings reveal extensive cooperation between thymic epithelial cells and multiple hematopoietic APC subsets in presentation of TRAs, suggesting a mechanism for the establishment of thymic central tolerance to rare self-antigens.

## Results

### Imaging selection of individual thymocytes to TRAs by 2PM

**Fig. 1:**
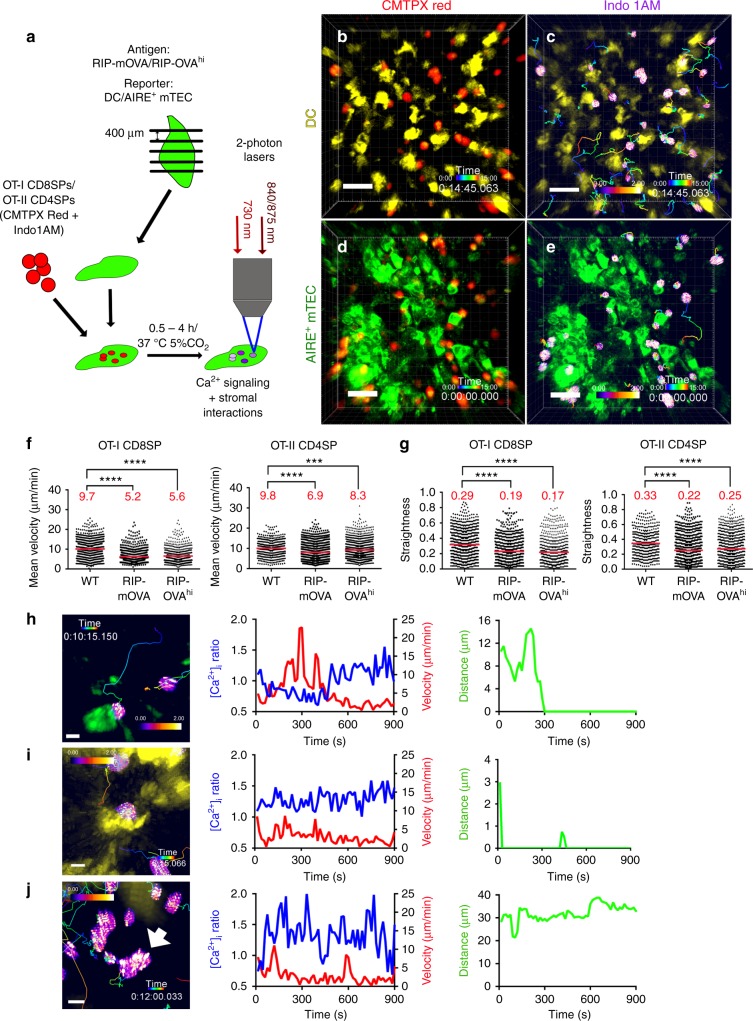
2PM approach for imaging antigen-specific SP thymocytes responding to TRAs presented by medullary APCs in live thymic slices. **a** CMTPX and Indo1AM-labeled OT-I CD8SPs or OT-II CD4SPs were incubated on live thymic slices expressing fluorescent reporters for DCs or AIRE^+^ mTECs, along with RIP-mOVA or RIP-OVA^hi^ transgenes. Time-lapse imaging through 40-μm depth was carried out with lasers tuned to excite Indo1AM (730 nm) and CMTPX with EGFP/EYFP (840/875 nm). **b**–**e** Maximum intensity projections of 2PM volumes, at ×40 magnification. SP thymocytes are displayed in **b**, **d** red or in **c**, **e** pseudo-color for [Ca^2+^]_i_ ratio, in **b**, **c** DC (yellow), or **d**, **e **AIRE^+^ mTEC (green) reporter slices. Thymocyte track times are color-encoded as indicated. Scale bar is 30 µm. **f** Mean cell velocity and **g** track straightness of individual OT-I CD8SP and OT-II CD4SP cells migrating in WT, RIP-mOVA, or RIP-OVA^hi^ slices. Data points represent cells from all experiments, and red bars and numbers show mean values. Data from 3 to 6 experiments per condition, number of cells: WT (*N*_OT-I_ = 827, *N*_OT-II _= 385), RIP-mOVA (*N*_OT-I_ = 780, *N*_OT-II _= 1138), and RIP-OVA^hi^ (*N*_OT-I_ = 738, *N*_OT-II _= 1354). Analyzed by *t* tests, *p*-values: *** < 0.001, **** < 0.0001. **h**–**j** Left: 2PM image of cells undergoing **h** initial activation, **i** sustained activation as a singlet, and **j** activation as an aggregate. Thymocyte tracks and Indo1 ratios are color-encoded. Scale bar is 5 µm. Middle: single-cell traces of [Ca^2+^]_i_ (blue) and velocity (red) over time. Right: cell distance to the nearest identified APC (DC or AIRE^+^ mTEC) over time. Data in **j** were from the thymocyte denoted by a white arrow. Source data are provided as a Source Data file. See also Supplementary Figs. 1, 2 and Movies 1–8

We developed a 2PM approach to visualize the migration and activation of SP thymocytes as they encounter negatively selecting ligands presented by AIRE^+^ mTECs or DCs in the thymic medulla (Fig. 1a). As a source of thymocytes expressing T-cell receptors (TCRs) of known specificities, we enriched MHC-I-restricted OT-I CD8SP or MHC-II-restricted OT-II CD4SP thymocytes from their respective strains^41,42^ on a *Rag2*^−/−^ background (Supplementary Fig. 1a). OT-I and OT-II TCRs bind ovalbumin (OVA) peptides in the context of H-2K^b^ and I-A^b^, respectively. To induce negative selection, thymocytes were overlaid on live thymic slices from RIP-mOVA or RIP-OVA^hi^ transgenic mice, which express OVA as a model TRA in transmembrane or secreted forms, respectively^43,44^. To visualize interactions with APCs, thymic slices also expressed a fluorescent reporter for DCs (Itgax-Venus^45^) (Fig. 1b–c; Supplementary Movie 1) or AIRE^+^ mTECs (*Aire*^EGFP^ ^9^ or *Adig*^46^) (Fig. 1d–e; Supplementary Movie 2). In this system, the mean velocities of OT-I CD8SPs and OT-II CD4SPs decreased significantly in RIP-mOVA and RIP-OVA^hi^ slices, compared with littermate wild-type (WT) slices (Fig. 1f; Supplementary Fig. 1b; Supplementary Movies 3–4), consistent with recognition of cognate antigens^47,48^. The mean path straightness of both OT-I and OT-II cells also decreased in RIP-mOVA and RIP-OVA^hi^ slices, indicating more tortuous migration upon self-antigen recognition (Fig. 1g).

While slower and more tortuous migration indicated that OT-I and OT-II cells were responding to OVA TRAs, the extensive overlap in cell velocities on WT versus RIP-OVA slices (Fig. 1f) precluded using low velocity to determine whether an individual SP cell was responding to self-antigens. Therefore, thymocytes were labeled with the ratiometric indicator dye Indo1AM^49^ to visualize changes in intracellular calcium concentrations ([Ca^2+^]_i_) as a proxy for TCR activation^50–53^. While previous imaging studies used exogenously administered peptides to induce simultaneous TCR signaling, thymocytes migrating in RIP-mOVA and RIP-OVA^hi^ slices asynchronously encounter OVA peptides presented by medullary APCs. Thus, we performed calibration experiments using exogenously added OVA peptides (OVAp) to define the minimum threshold, above which the average Indo1AM emission ratio must increase for a thymocyte to be considered activated (Supplementary Fig. 1c). Using these criteria, we classified two types of activated thymocytes: those undergoing initial activation, in which [Ca^2+^]_i_ increased acutely from baseline levels during imaging (Fig. 1h; Supplementary Movie 5), and those with sustained activation, in which high [Ca^2+^]_i_ was sustained throughout imaging (Fig. 1i; Supplementary Movie 6) due to initiation of TCR signaling prior to image acquisition. Activated thymocytes were observed both as single cells (Fig. 1h) and as thymocyte aggregates (Fig. 1j; Supplementary Movie 7). Thus, using 2PM imaging, we are able to discern activation of individual thymocytes in live thymic slices.

We also quantified the distance between thymocytes and AIRE^+^ mTECs (Fig. 1h) or DCs (Fig. 1i), in their respective reporter slices, to determine whether activated thymocytes were contacting these APCs. For example, in Fig. 1h, as the thymocyte contacts an AIRE^+^ mTEC, its [Ca^2+^]_i_ increases and velocity declines, indicating that the AIRE^+^ mTEC presented an antigen to activate this thymocyte. In Fig. 1i, high [Ca^2+^]_i_ and low velocity are sustained as the activated thymocyte stably contacts a DC throughout imaging. Notably, some activated thymocytes are not in contact with a visible APC (Fig. 1j). Thus, the density of DCs and AIRE^+^ mTECs in the medulla is not so high as to lead us to falsely conclude that thymocytes undergoing TCR activation are in contact with whichever APC type expresses the fluorescent reporter. Since TCR activation requires antigen presentation by APCs, activated cells that are not in contact with a visible APC are likely in contact with the other major non-labeled APC subset. To test this, we carried out imaging experiments on dual-reporter CD11c-mCherry^54^ × *Aire*^*E*GFP^ thymic slices, in which the addition of OVAp was used to activate OT-I and OT-II thymocytes (Supplementary Fig. 2a, b; Supplementary Movie 8). Nearly all (98%) of the activated thymocytes contacted either AIRE^+^ mTECs or DCs (Supplementary Fig. 2c), indicating that these are the two predominant APC subsets that present antigens to induce thymocyte tolerance in this system. These results also support our conclusion that activated thymocytes not in contact with a visible APC in single-reporter slices are most likely contacting the other major APC subsets. Furthermore, even in regions dense with DCs and AIRE^+^ mTECs, activated thymocytes unambiguously contact either DCs or AIRE^+^ mTECs (Supplementary Fig. 2c), validating our conclusion that when an activated cell contacts a visible APC in single-reporter slices, it is recognizing a peptide presented by that APC. Thus, our 2PM imaging approach allows us to detect thymocytes undergoing signaling and to determine if they are in contact with AIRE^+^ mTECs or DCs.

### OT-I and OT-II SP respond with distinct efficiencies to TRAs

**Fig. 2:**
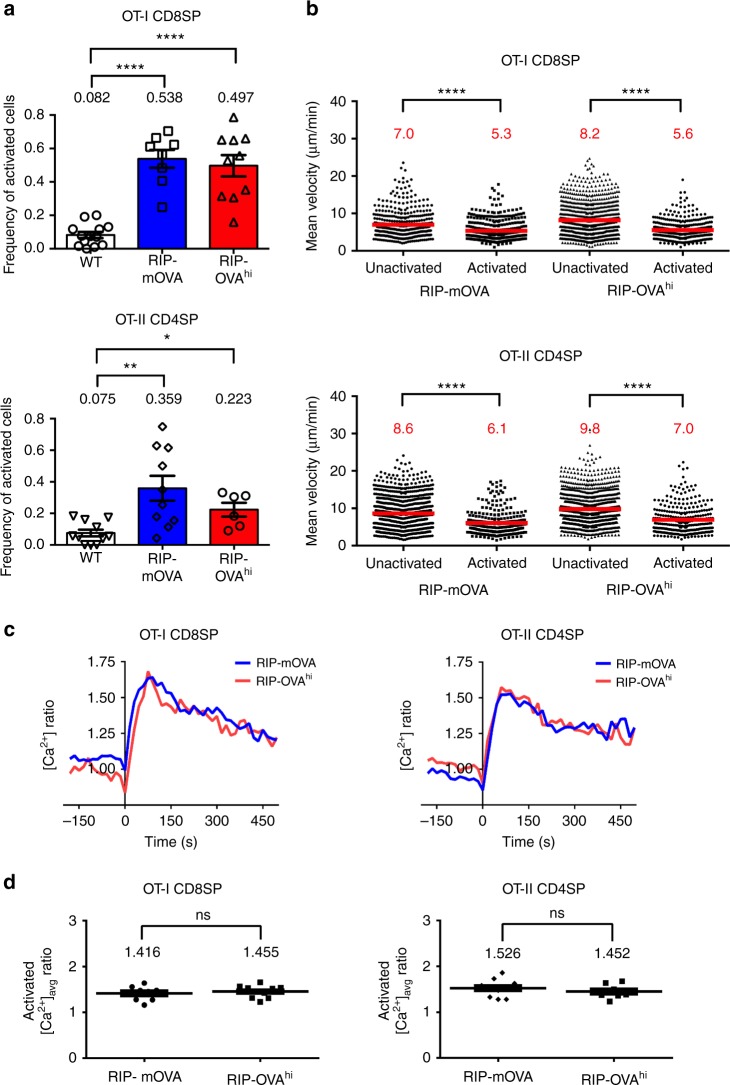
The frequency of activation, but not the magnitude of calcium flux, differs for OT-I versus OT-II thymocytes responding to distinct model TRAs. **a** Frequencies of OT-I CD8SP and OT-II CD4SP thymocytes activated in WT (white), RIP-mOVA (blue), or RIP-OVA^hi^ (red) thymic slices. Data points represent the average of all cells in an individual experiment, and bars represent mean ± standard error of the mean (SEM). **b** Individual mean cell velocities of unactivated and activated OT-I CD8SPs and OT-II CD4SPs migrating on RIP-mOVA or RIP-OVA^hi^ thymic slices. Data points represent individual cells in all experiments, and red bars and numbers show mean values. **c** Average [Ca^2+^]_i_ levels of OT-I CD8SPs and OT-II CD4SPs undergoing initial activation on RIP-mOVA (blue; *N*_OT-I_ = 35, *N*_OT-II _= 23) or RIP-OVA^hi^ (red; *N*_OT-I_ = 19, *N*_OT-II _= 18) thymic slices were quantified and traced across imaging experiments. Single-cell calcium traces were aligned with the lowest ratio value before flux (as defined in the Methods section) set at time point 0. **d** The average calcium ratios of all activated OT-I CD8SPs and OT-II CD4SPs on RIP-mOVA or RIP-OVA^hi^ thymic slices were quantified. Data points represent the average of all cells in an experiment, and bars and whiskers represent mean ± SEM. Data are compiled from 3 to 6 separate imaging experiments per condition. Analyzed by unpaired *t* tests, *p*-values: * < 0.05, ** < 0.01, **** < 0.0001. ns not significant. Source data are provided as a Source Data file. See also Supplementary Fig. 3

To confirm that thymocytes classified as activated on the basis of increased [Ca^2+^]_i_ were undergoing TCR-mediated signaling, we compared the frequencies of activated OT-I CD8SPs and OT-II CD4SPs detected in medullary regions of RIP-mOVA, RIP-OVA^hi^, and WT thymic slices. OT-I and OT-II cells were activated significantly more frequently in OVA-expressing slices, indicating that activation reflected TCR-mediated signaling in response to cognate antigens (Fig. 2a). Approximately half of the OT-I CD8SP thymocytes were activated in RIP-mOVA and RIP-OVA^hi^ slices (Fig. 2a). One-third of OT-II CD4SP thymocytes were activated in RIP-mOVA slices, and about one-quarter were activated in RIP-OVA^hi^ slices (Fig. 2a). Activated OT-I and OT-II cells migrated more slowly than unactivated cells on OVA slices (Fig. 2b), indicative of TCR activation-induced thymocyte arrest^47,53^. The activated thymocytes also migrated more tortuously (Supplementary Fig. 3a), suggesting that they stay in proximity to the APC to which they respond for prolonged periods. Rare OT-I and OT-II cells with elevated [Ca^2+^]_i_ and reduced velocity were also detected on WT thymic slices (Fig. 2a; Supplementary Fig. 3b), consistent with previous findings from our lab and others that OT-II CD4SP thymocytes undergo low-level deletion in response to endogenous self-antigens in C57BL/6 thymi^14,55,56^. Altogether, detection of an antigen by 22–54% of OT-I and OT-II cells within 2 h of migration in RIP-mOVA and RIP-OVA^hi^ slices demonstrates that thymocytes efficiently encounter TRAs in situ.

We next analyzed single-cell calcium traces to determine whether transmembrane versus secreted TRAs or presentation on MHC-I versus MHC-II resulted in qualitative differences in signaling following TCR activation. Given the comparable changes in [Ca^2+^]_i_ for OT-I and OT-II cells undergoing initial activation on RIP-mOVA and RIP-OVA^hi^ slices (Fig. 2c), and the comparable average [Ca^2+^]_i_ for all activated thymocytes (Fig. 2d), we found no evidence for different levels of TCR signaling for OT-I or OT-II cells responding to OVA TRAs. Thus, the lower activation frequency of OT-II cells in RIP-OVA^hi^ versus RIP-mOVA slices (Fig. 2a) likely reflects that a lower frequency of OT-II cells encountered sufficient antigen to induce activation in the case of the secreted OVA TRA. Taken together with the findings that OT-I CD8SPs are activated more frequently than OT-II CD4SPs, despite comparable affinities of the OT-I and OT-II TCRs for OVAp:MHC complexes^57,58^, these data indicate that the combination of the cellular compartment in which the TRA is expressed and the MHC class on which peptides are presented can alter antigen availability and thus the frequency of TCR activation events.

### SPs activated by TRAs in thymic slices undergo selection

**Fig. 3:**
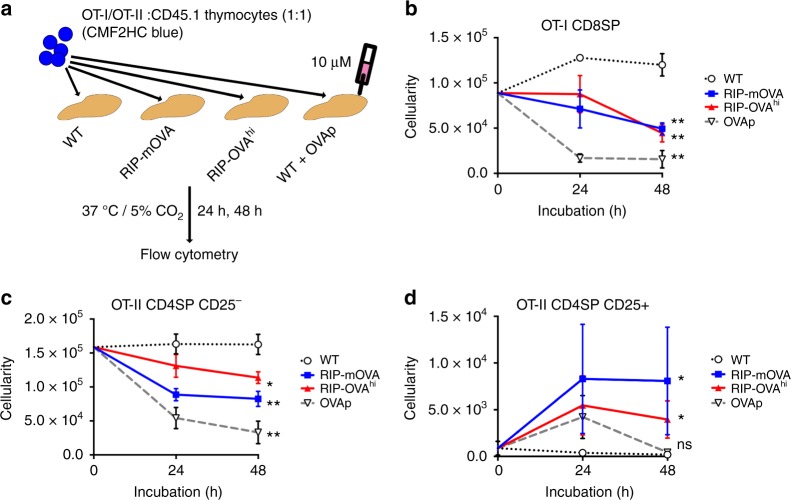
Activated OT-I and OT-II thymocytes in RIP-mOVA and RIP-OVA^hi^ slices predominantly undergo negative selection. **a** Schematic of slice deletion assays: OT-I or OT-II thymocytes were combined (1:1) with CD45.1 thymocytes, all of which were labeled with CMF2HC Blue and incubated on WT, RIP-mOVA, or RIP-OVA^hi^ thymic slices. As a positive control for deletion, 10 µM of the cognate OVA peptide (OVAp) for OT-I or OT-II TCRs was added exogenously to some WT slices. Number of **b** OT-I CD8SP, **c** OT-II CD4SP CD25^−^, and **d** OT-II CD4SP CD25^+^ thymocytes in WT (white circle), RIP-mOVA (blue), RIP-OVA^hi^ (red), and OVAp slices (white triangle) at the indicated time points by flow cytometry. Plots show mean ± SEM of compiled data from three independent OT-I, and four independent OT-II experiments, each with triplicate thymic slices per condition. Analyzed by one-way ANOVA between WT and the indicated OVA conditions at 48 h, with Bonferroni correction for multiple comparisons, *p*-values: * < 0.05, ** < 0.01. ns not significant. Source data are provided as a Source Data file. See also Supplementary Fig. 4 for flow-cytometric gating schemes

To determine whether activation of OT-I CD8SP and OT-II CD4SP thymocytes in RIP-mOVA and RIP-OVA^hi^ slices resulted in clonal deletion or Treg induction, we utilized a thymic slice deletion assay^50,55,56^ (Fig. 3a). OVAp was added to WT thymic slices containing OT-I or OT-II cells as a positive control for tolerance induction. Flow-cytometric analysis (Supplementary Fig. 4) revealed that OT-I and OT-II cells are in a highly competitive environment with polyclonal thymocytes in thymic slices, representing ~0.05% of the cells. The number of OT-I CD8SP and OT-II CD4SP CD25^−^ thymocytes declines significantly after 48 h in RIP-mOVA and RIP-OVA^hi^ slices when compared with WT slices, demonstrating that clonal deletion occurs in response to endogenous TRAs (Fig. 3b–c). The extent of deletion is roughly proportional to the frequency of activated OT-I and OT-II cells on RIP-mOVA or RIP-OVA^hi^ slices (Fig. 2a). Differentiation of OT-II cells into CD4^+^CD25^+^ Treg precursors is induced in RIP-mOVA and RIP-OVA^hi^ slices (Fig. 3d), but represents a rare event. Altogether, negative selection is the predominant outcome for OT-I and OT-II cells activated by OVA TRAs in thymic slices.

### Relative contributions of AIRE^+^ mTECs and DCs to selection

**Fig. 4:**
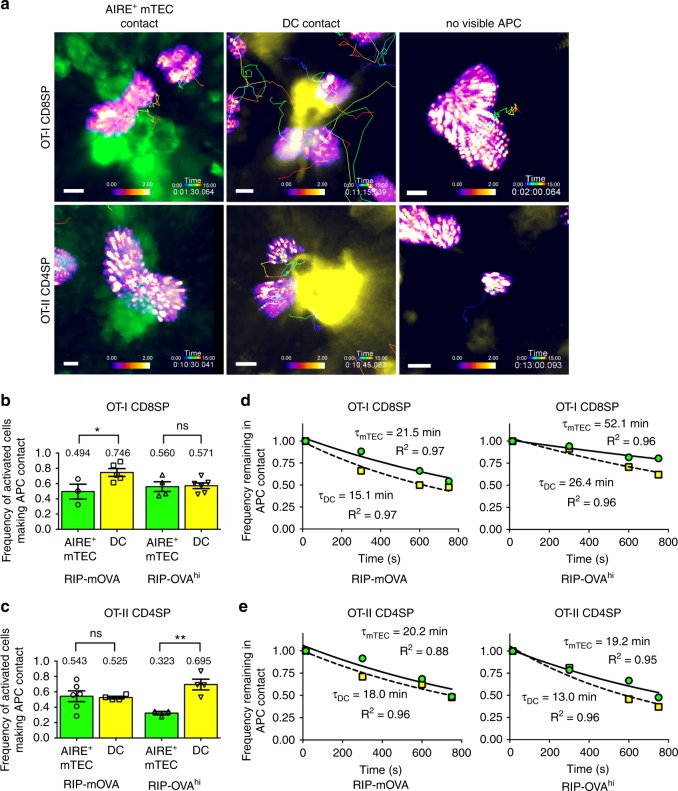
AIRE^+^ mTECs and DCs contribute differentially to activation of OT-I and OT-II SPs undergoing negative selection in RIP-mOVA versus RIP-OVA^hi^ slices. **a** Representative 2PM images of activated OT-I CD8SP and OT-II CD4SP thymocytes contacting AIRE^+^ mTECs, DCs, or no visible APC. Thymocyte track time is color-encoded. Scale bar is 5 µm. **b**, **c** Frequency of activated **b** OT-I CD8SPs and **c** OT-II CD4SPs interacting with AIRE^+^ mTECs or DCs on thymic slices expressing RIP-mOVA or RIP-OVA^hi^ TRAs,  from *Adig* (green) or CD11c-EYFP (yellow) reporter mice. Data points represent the average of all cells in an experiment, and bars show mean ± SEM. Data from 3 to 6 experiments per condition, number of cells: RIP-mOVA (*N*_OT-I_ = 371, *N*_OT-II _= 265), RIP-OVA^hi^ (*N*_OT-I_ = 301, *N*_OT-II _= 283). Analyzed by unpaired *t* tests, *p*-values: * < 0.05, ** < 0.01. ns not significant. **d**, **e** The frequencies of activated **d** OT-I CD8SPs and **e** OT-II CD4SPs that remain in contact with their activating AIRE^+^ mTEC (green) or DC (yellow) for the 15-min imaging duration on RIP-mOVA or RIP-OVA^hi^ thymic slices were fit to a one-phase exponential decay. Nonlinear fit shown for interaction with mTEC (solid line) and DC (dashed line), mean lifetime τ, and goodness of fit *R*^2^ as indicated. Data are compiled from 3 to 6 experiments per condition, number of cells: RIP-mOVA (*N*_OT-I_ = 148, *N*_OT-II _= 104), RIP-OVA^hi^ (*N*_OT-I_ = 146, *N*_OT-II _= 104). Source data are provided as a Source Data file. See also Supplementary Figs. 5, 6

We next quantified the frequency with which activated OT-I CD8SP and OT-II CD4SP thymocytes contacted AIRE^+^ mTECs or DCs during tolerance induction in RIP-mOVA and RIP-OVA^hi^ thymic slices. OT-I and OT-II cells were observed contacting AIRE^+^ mTECs or DCs as they underwent activation (Fig. 4a). Some activated thymocytes were not in contact with fluorescently labeled stromal cells, presumably because they were interacting with the non-labeled APC subset (Fig. 4a), as discussed above and supported by dual-reporter imaging (Supplementary Fig. 2). Activated OT-I CD8SPs contacted DCs slightly more frequently than they contacted AIRE^+^ mTECs in RIP-mOVA slices (Fig. 4b). In RIP-OVA^hi^ slices, DCs and AIRE^+^ mTECs activated OT-I cells with roughly equal frequencies (Fig. 4b). OT-II CD4SP thymocytes interacted with DCs and AIRE^+^ mTECs with equal frequencies as they underwent activation in RIP-mOVA slices (Fig. 4c). However, in RIP-OVA^hi^ slices, activated OT-II CD4SPs contacted DCs significantly more frequently than AIRE^+^ mTECs (~70% vs. 30%) (Fig. 4c). Thus, AIRE^+^ mTECs and DCs both contribute to the activation of OT-I and OT-II thymocytes responding to transmembrane and secreted forms of the OVA TRA, with DCs activating OT-I and OT-II thymocytes more frequently in RIP-mOVA and RIP-OVA^hi^ slices, respectively.

The relative contributions of AIRE^+^ mTECs versus DCs to activation of OT-I CD8SP and OT-II CD4SP cells were comparable, regardless of whether we limited our analyses to thymocytes undergoing initial activation (Supplementary Fig. 5a), or included thymocytes undergoing initial and sustained activation events (Fig. 4b–c). This analysis confirms the extensive cooperation between AIRE + mTECs and DCs in presenting TRAs to initiate negative selection of OT-I CD8SPs and OT-II CD4SPs, with a greater role for DCs in activating OT-II CD4SP cells responding to the RIP-OVA^hi^ TRA. We considered the possibility that the relative cellularity of DCs versus AIRE^+^ mTECs could dictate how frequently each of them interacted with thymocytes. Our data indicate that this is not the case: thymic DCs are more abundant than AIRE^+^ mTECs (Supplementary Fig. 5b), as quantified by flow cytometry of WT, RIP-mOVA, and RIP-OVA^hi^ mice (Supplementary Fig. 5c). However, AIRE^+^ mTECs activate OT-I CD8SP cells and OT-II CD4SP as frequently as DCs in RIP-OVA^hi^ and RIP-mOVA thymi, respectively (Fig. 4b–c). Altogether, while both AIRE^+^ mTECs and DCs contribute to negative selection of OT-II CD4SPs, DCs play a greater role in the context of a secreted TRA, indicating that the cellular compartment in which a TRA is expressed impacts the efficiency of presentation by mTECs versus DCs. The finding that DCs in RIP-OVA^hi^ mice contributed more frequently to antigen presentation to OT-II versus OT-I SP cells, indicates that processing and presentation on MHC-I versus MHC-II also impact the efficiency of presentation by mTECs and DCs.

To determine whether the duration of thymocyte contacts differed for the two APC subsets, we estimated mean lifetimes (τ) of thymocyte–APC interactions under each condition (Fig. 4d–e). On RIP-mOVA slices, ~50% of activated OT-I CD8SP and OT-II CD4SP cells disengage from their APCs during the 15-min imaging duration, with mean lifetimes in the range of 15–20 min (Fig. 4d–e). On RIP-OVA^hi^ slices, the mean lifetimes of interaction between OT-I CD8SPs and both types of APCs were longer, in the range of 25–50 min, with nearly 75% of the cells remaining in contact with their respective APCs throughout imaging (Fig. 4d–e). These data demonstrate that both APC types are capable of forming long-lasting contacts with thymocytes (≥ 25 min) during tolerance induction, so the duration of the interaction is not determined by the APC type alone. Interestingly, OT-II thymocytes had the shortest mean lifetime of interactions when contacting DCs on RIP-OVA^hi^ slices, ~13 min, such that only ~25% of thymocytes remained in contact at the end of imaging. Thus, although DCs play a greater role than AIRE^+^ mTECs in activating OT-II thymocytes in RIP-OVA^hi^ slices (Fig. 4c), they may not present this secreted TRA very efficiently, resulting in less prolonged contacts, less efficient activation (Fig. 2a), and less efficient negative selection (Fig. 3c).

To determine the extent to which antigen acquisition from mTECs and cross-presentation on MHC-I contributed to DC-mediated activation of OT-I CD8SP cells, we investigated whether expression of the *Ova* TRA was restricted to mature mTECs, as expected, in these *Ova* transgenic strains. *Ova* transcripts are detected in multiple medullary APC subsets (Supplementary Fig. 6a) sorted from RIP-mOVA mice (Supplementary Fig. 6b). A recent study revealed that antisense *Ova* transcripts could account for this unexpected expression pattern. In this study, in situ hybridization for sense *Ova* transcripts revealed expression by sparse medullary cells^59^, consistent with expression by mature mTECs. However, we cannot rule out that OVA is expressed by multiple medullary APCs, including DCs, in RIP-mOVA mice, and thus cannot conclude whether cross-presentation occurs using this model. In contrast, in RIP-OVA^hi^ mice, *Ova* transcripts are detected only in MHC-II^hi^ mTECs (mTEC^hi^) (Supplementary Fig. 6a). Despite the fact that DCs do not express *Ova* transcripts, they activate OT-I CD8SP cells as frequently as mature mTECs themselves in RIP-OVA^hi^ thymi (Fig. 4b). Thus, relative expression levels of TRAs by AIRE^+^ mTECs versus DCs are not sufficient to account for the relative contributions of these APCs to thymocyte activation. In RIP-OVA^hi^ thymi, DCs efficiently acquire OVA expressed by mTEC^hi^ cells and cross-present peptides on MHC-I, contributing as significantly to negative selection of CD8SP cells as AIRE^+^ mTECs.

2PM also revealed that activated OT-II CD4SP thymocytes formed aggregates more frequently than activated OT-I CD8SP thymocytes (Supplementary Fig. 6c). The formation of antigen-specific T-cell clusters with high intracellular calcium levels and reduced motility has been reported in live-cell imaging studies within both the lymph node^60^ and the thymus^48,47^, and suggests that the APC nucleating the aggregate is particularly efficient at presenting an antigen. In contrast to previous findings^48^, AIRE^+^ mTECs and DCs contributed roughly equally to aggregate formation with thymocytes undergoing tolerance (Supplementary Fig. 6d), indicating that the ability to nucleate thymocyte aggregates is not a unique feature of one APC type.

### Both cDC subsets contribute to TRA-induced selection

**Fig. 5:**
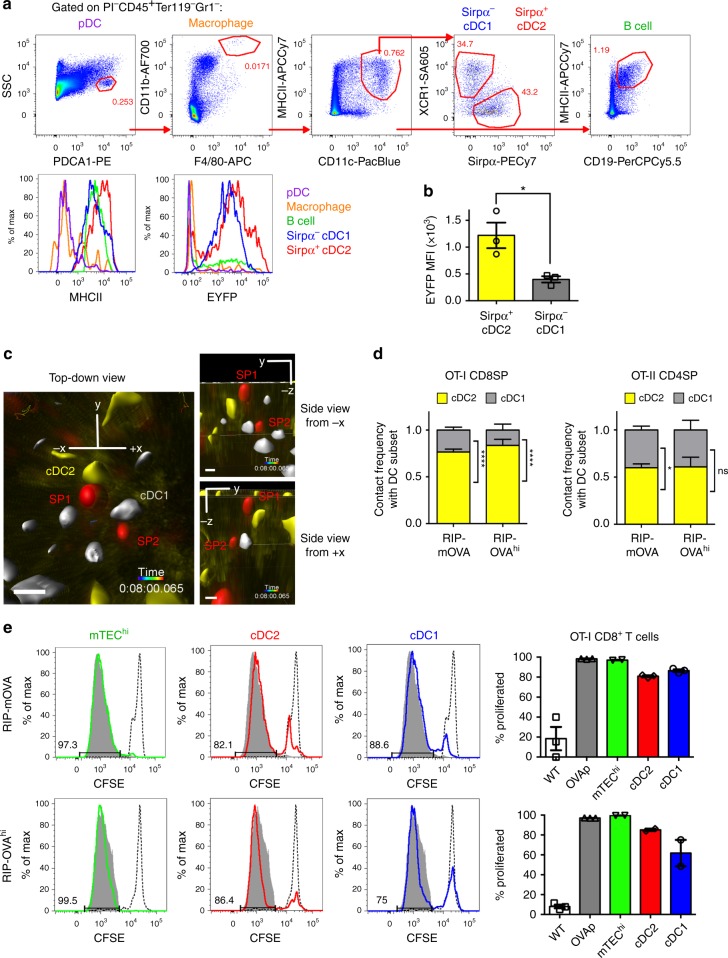
Both conventional DC subsets induce negative selection of OT-I and OT-II SPs responding to RIP-mOVA and RIP-OVA^hi^ TRAs. **a** Sequential gating of live CD45^+^ cells from a digested CD11c-EYFP thymus to detect the indicated APC subsets. Histograms show EYFP and MHC-II levels for the indicated APC subsets. **b** EYFP mean fluorescence intensity (MFI) of Sirpα^+^ cDC2 and Sirpα^−^ cDC1. Flow-cytometry data averaged from three CD11c-EYFP mice, stained independently. Data points represent mice, and bars show mean ± SEM. **c** 2PM volume from three perspectives, showing two activated OT-I CD8SPs (red mask, SP1 and SP2) interacting with cDC2 (yellow) and cDC1 (gray), respectively. Scale bar is 10 µm. **d** Frequency of activated OT-I CD8SP and OT-II CD4SP thymocytes contacting DCs, that interacted with cDC2 or cDC1 on RIP-mOVA or RIP-OVA^hi^ thymic slices. Graph shows mean + SEM. Data are compiled from experiments analyzed in Fig. 4. Analyzed by *t* tests with multiple comparison correction, *p*-values: * < 0.05, *** < 0.001. ns not significant. **e** mTEC^hi^ (green), cDC2 (red), and cDC1 (blue) were sorted from RIP-mOVA and RIP-OVA^hi^ thymi and cultured with CFSE-labeled splenic OT-I CD8^+^ T cells. WT splenocytes ± OVAp served as positive and negative control APCs. Histograms show CFSE dilution in Vα2^+^Vβ5^+^CD8^+^ cells after incubation with APCs for 72 h, the gate shows the percent of cells that proliferated. Dashed line shows the CFSE profile for T cells cultured with WT splenocytes in the absence of OVAp, and gray shading shows that of T cells cultured with OVAp-pulsed WT splenocytes. Data are representative of two independent experiments per condition, and graphs depict mean ± SEM of the percent proliferation after incubation with indicated APCs for triplicate wells. Source data are provided as a Source Data file. See also Supplementary Figs. 7, 8

Contact with DCs or AIRE^+^ mTECs accounted for 100% of OT-I and OT-II SP activation in both RIP-mOVA and RIP-OVA^hi^ slices (Fig. 4b–c), suggesting negligible contributions from other APC subsets to negative selection in this system. Nonetheless, other hematopoietic APCs, such as B cells, macrophages, or pDCs could potentially participate in negative selection against OVA TRAs. To address this possibility, we analyzed MHC-II on thymic APCs by flow cytometry in CD11c-EYFP thymi. Neither plasmacytoid dendritic cells (pDCs) nor CD11b^hi^F4/80^hi^ macrophages expressed high levels of MHC-II or EYFP (Fig. 5a), indicating that they did not contribute to negative selection of OT-II CD4SPs and were not mistaken for DCs contacting activated thymocytes in CD11c-EYFP reporter slices. Thymic B cells expressed high levels of MHC-II, as previously reported^61,62^, and ~20% were EYFP^+^ (Fig. 5a). Thus, we investigated their role in negative selection by generating µMT-deficient OT-II bone chimeras. We did not detect a requirement for B cells in negative selection of OT-II thymocytes responding to the RIP-OVA^hi^ TRA in vivo (Supplementary Fig. 7a, b), similar to a previous finding in RIP-mOVA recipients^59^. However, B-cell deficiency did result in a significant increase in OT-II CD4SP cellularity in the absence of the OVA TRA, suggesting that B cells may promote deletion of OT-II thymocytes to an endogenous self-antigen. Altogether, these data indicate that antigen presentation by DCs and AIRE^+^ mTECs accounts for the vast majority of APC activity responsible for negative selection in RIP-mOVA and RIP-OVA^hi^ thymi, and that contact of activated thymocytes with EYFP^+^ cells in CD11c-EYFP slices reflects interactions with DCs.

Conventional thymic DCs can be subdivided into two major subsets based on SIRP*α* expression^63^, and both subsets express EYFP and MHC-II in CD11c-EYFP thymi (Fig. 5a). Sirpα^+^ DCs (cDC2)^40^ express approximately threefold higher levels of EYFP compared with Sirpα^−^ DCs (cDC1) (Fig. 5b). Using a threshold mask based on the relative EYFP intensities from flow-cytometric data, EYFP^hi^ versus EYFP^lo^ DCs could be distinguished in 2PM data (Fig. 5c). Using this approach, we quantified the frequency with which Sirpα^+^ (EYFP^hi^) cDC2 versus Sirpα^−^ (EYFP^lo^) cDC1 presented antigens to induce tolerance. Activated OT-I CD8SP thymocytes contact cDC2 significantly more frequently than cDC1 in both RIP-mOVA and RIP-OVA^hi^ slices (Fig. 5d), indicating efficient cross-presentation of TRAs by cDC2. In contrast, activated OT-II CD4SP cells contacted cDC1 and cDC2 with approximately equal frequencies in both TRA models (Fig. 5d). Previous studies suggested that cDC1 were particularly critical for acquiring and cross-presenting TRAs expressed by mTECs^25,27,31^. To confirm that cDC2 could cross-present the mTEC-restricted RIP-OVA^hi^ TRA on MHC-I, as well as to confirm that AIRE^+^ mTECs, cDC1, and cDC2 presented both TRA forms, as indicated by our imaging studies, we assessed the capacity of FACS-purified APCs from RIP-mOVA and RIP-OVA^hi^ thymi (Supplementary Fig. 8a) to induce proliferation of OT-I CD8^+^ T cells (Supplementary Fig. 8b). Consistent with our imaging results, cDC1, cDC2, and mTEC^hi^ all induced OT-I T-cell proliferation (Fig. 5e), demonstrating that all of these APCs displayed OVA  peptides on MHC-I in both TRA strains. These data indicate that cDC2 are more efficient than cDC1 at acquiring and cross-presenting some *Aire*-dependent TRAs.

### Polyclonal SPs are activated by both AIRE^+^ mTECs and DCs

**Fig. 6:**
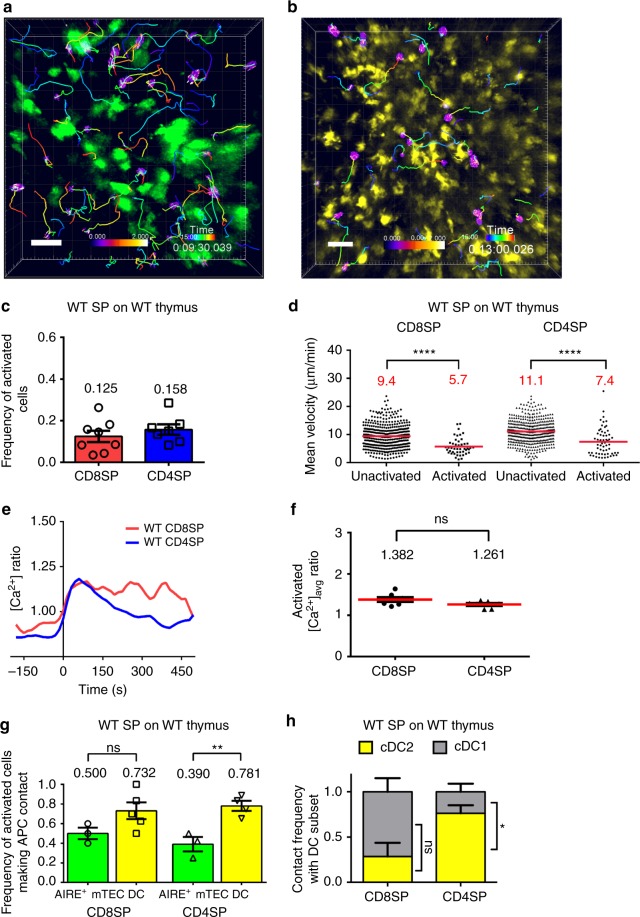
Both AIRE^+^ mTECs and DCs engage polyclonal SP thymocytes undergoing TCR signaling. **a**, **b** Maximum intensity projections of 2PM imaging volumes collected at ×20 magnification showing **a** WT CD4SP thymocytes (Indo1 ratio) migrating in *Aire*^EGFP^ (green) or **b** WT CD8SP thymocytes (Indo1 ratio) migrating in CD11c-EYFP (yellow) slices. Thymocyte tracks are color-encoded for time; scale bar is 30 µm. **c** Frequency of polyclonal CD8SP (red) and CD4SP (blue) thymocytes undergoing activation in WT thymic slices. Data points represent the average of all cells in an experiment, and bars show mean ± SEM. **d** Mean cell velocities of unactivated and activated WT CD8SPs and CD4SPs in WT thymic slices. Data points represent individual cells in all experiments, and the red bar and numbers show means. **e** Average [Ca^2+^]_i_ of WT CD8SP (red; *N* = 3) and CD4SP thymocytes (blue; *N* = 19) undergoing initial activation on WT thymic slices. Single-cell calcium traces were aligned such that the lowest ratio value before the increase in [Ca^2+^]_i_ was set at time point 0. **f** Average [Ca^2+^]_i_ of all activated WT CD8SPs and CD4SPs on WT thymic slices. Data points represent the average of all cells in an experiment, and bars show mean ± SEM. **g** Frequency of activated WT CD8SP and CD4SP thymocytes contacting AIRE^+^ mTECs or DCs on *Aire*^EGFP^ (green) or CD11c-EYFP (yellow) thymic slices. Data points represent the average of all cells in an experiment, and bars show mean ± SEM. **h** Frequency of activated WT CD8SPs and CD4SPs contacting DCs, that interacted with cDC2 (yellow) or cDC1 (gray) on CD11c-EYFP thymic slices. Graphs show mean + SEM. Data compiled from 7 to 8 separate imaging experiments per condition, number of cells: CD8SP (*N*_unactivated_ = 399, *N*_activated _= 46), CD4SP (*N*_unactivated_ = 419, *N*_activated _= 60). Analyzed by unpaired *t* tests, except for **h** which was analyzed by *t* tests with Sidak–Bonferroni correction for multiple comparisons, *p*-values: * < 0.05, ** < 0.01, **** < 0.0001, ns: not significant. Source data are provided as a Source Data file. See also Supplementary Movies 9, 10

Our studies of TCR transgenic thymocytes responding to model TRAs indicate that both AIRE^+^ mTECs and DCs can contribute efficiently to negative selection of some thymocyte clones restricted to MHC-I and MHC-II. To determine whether these findings were more broadly representative of selection of the polyclonal repertoire, we imaged polyclonal CD8SPs and CD4SPs undergoing TCR signaling in response to endogenous self-antigens (Fig. 6a–b; Supplementary Movies 9–10). A small frequency of CD4SPs (12.8%) and CD8SPs (15.8%) displayed elevated [Ca^2+^]_i_ in WT thymic slices (Fig. 6c). The activated cells exhibited reduced velocities compared with unactivated cells (Fig. 6d). Activation of polyclonal thymocytes was less frequent than that previously reported in a 2PM imaging study that relied on reduced thymocyte motility alone to identify cells undergoing activation^47^, consistent with our observation that low velocity is not sufficient to discriminate activated thymocytes (Fig. 2b). The change in [Ca^2+^]_i_ for thymocytes undergoing initial activation was similar for CD8SPs and CD4SPs (Fig. 6e), and the average elevation in [Ca^2+^]_i_ in activated thymocytes was comparable for CD8SPs and CD4SPs (Fig. 6f). Thus, rare polyclonal cells could be observed undergoing TCR signaling in live thymic slices.

Similar to OT-I cells, WT CD8SP thymocytes interacted approximately equally with AIRE^+^ mTECs and DCs as they underwent activation (Fig. 6g). In contrast, activated WT CD4SPs interacted significantly more frequently with DCs (Fig. 6g), as was the case for OT-II CD4SP thymocytes in RIP-OVA^hi^ slices. Despite the greater contribution of cDC2 to negative selection of OT-I CD8SPs, cDC1 contributed at least as frequently to interactions with activated polyclonal CD8SPs (Fig. 6h). cDC2 interacted more frequently than cDC1 with activated polyclonal CD4SPs (Fig. 6h), similar to the results with OT-II CD4SPs. Thus, the relative contribution of mTECs and DCs to selection of a polyclonal repertoire largely mirrored our findings with OT-I and OT-II TCR transgenic thymocytes responding to TRAs, although the relative contribution of DC subsets diverged, with increased interactions of polyclonal CD8SPs with cDC1.

## Discussion

Although the intrinsic capacity of both AIRE^+^ mTECs^13,15,17^ and DCs^14,16,23^ to present TRAs to induce negative selection has been documented, prior experimental strategies could not differentiate the relative contributions of these medullary APC subsets when both were present and functional. The 2PM imaging approach described here enables real-time visualization of thymocyte:APC interactions in relation to TCR signaling, allowing us to monitor which APC subsets induce TCR activation under negatively selecting conditions. Previous 2PM studies of negative selection in thymic slices were limited to observing thymocyte motility, concurrently with either APC interactions^47,50^ or TCR activation alone^51^. Importantly, we observed interactions between thymocytes and APCs that did not result in TCR activation, indicating that contact with APCs is not sufficient for identification of thymocytes undergoing tolerance induction. Furthermore, earlier studies investigated calcium-signaling dynamics of negative selection in post-positive selection CD4^+^CD8^+^ thymocytes responding to exogenously added peptides^50,51^. In contrast, we imaged CD8SPs and CD4SPs undergoing negative selection in response to TRAs in the thymic medulla, which is a more physiologically relevant system for studying tolerance against rare medullary self-antigens.

Using this approach, we found that AIRE^+^ mTECs and DCs contributed roughly equally to activation of OT-I CD8SPs undergoing negative selection against transmembrane and secreted OVA TRAs. Surprisingly, cDC2 cross-presented mTEC-derived TRAs to OT-I CD8SPs significantly more than cDC1. For OT-II CD4SPs, interactions with AIRE^+^ mTECs and DCs occurred with equal frequency during negative selection against the transmembrane RIP-mOVA TRA, with roughly equal contributions by cDC1 and cDC2. However, when OVA was expressed as a secreted TRA, DCs contributed significantly more than mTECs to negative selection, accounting for greater than two-thirds of the activation events in RIP-OVA^hi^ slices. Consistent with these imaging results, bone marrow chimeras revealed that antigen presentation by hematopoietic APCs is required for negative selection of OT-II CD4SPs in RIP-OVA^hi^ hosts^14^. Furthermore, our studies show that both MHC restriction and the subcellular localization of TRAs modulated the relative contributions of thymic APCs to negative selection. The frequencies with which AIRE^+^ mTECs and DCs contributed to negative selection of monoclonal OT-I and OT-II cells were largely mirrored in the polyclonal setting, where activation of polyclonal thymocytes reflects signaling in response to both transmembrane and secreted self-antigens. In both the polyclonal and monoclonal settings, DCs contributed more frequently to activation of CD4SPs than AIRE^+^ mTECs. We have previously demonstrated that the chemokine receptor CCR4, which is expressed by CD4SP thymocytes, promotes thymocyte interactions with DCs and negative selection^55^. Thus, it will be interesting to further investigate whether differential expression of chemokine receptors impacts the efficiency with which different thymocyte subsets interact with APCs to undergo tolerance.

There are notable differences in the conclusions that can be reached using 2PM to directly visualize APC subsets that induce negative selection, versus using TCR repertoire sequencing, in which genetic models are employed to ablate one APC subset or impair its ability to present antigens, to deduce the requirement for mTECs or DCs for selection of TCR clones^27,28,31^. Imaging can reveal the relative contribution of APC subsets to antigen presentation even if both APC types contribute equally. By design, the TCR repertoire-sequencing approaches identify a requirement for an APC subset in selection only if its absence significantly alters the frequency of a TCR clone, which would not be the case if multiple APC subsets are sufficient to select a given clone. Thus, repertoire-sequencing studies have revealed that mTECs and DCs select some distinct TCR clones^27,28,31^, demonstrating that they present distinct self-antigens. In contrast, our findings demonstrate that thymocytes expressing a monoclonal TCR can encounter the same antigen presented by both major APC subsets, indicating an overlap in the pool of self-antigens displayed by mTECs and DCs. In this regard, direct visualization by 2PM can show that both APC subsets contribute to negative selection, potentially resolving some seemingly disparate studies of model OVA antigens that concluded that either mTECs^13,14,16^ or DCs^14,16,23^ were responsible for tolerance induction.

A recent study highlighted the capacity of cDC1 to efficiently acquire antigens from mTECs for cross-presentation to CD8SP thymocytes^25^. However, this study did not directly compare the abilities of cDC1 versus cDC2 to present mTEC-derived antigens. We find that cDC2 cross-present OVA to a greater percentage of activated OT-I CD8SP thymocytes, even in the context of mTEC-restricted RIP-OVA^hi^. This finding is consistent with a previous study, showing that thymic cDC2 can cross-present antigens on MHC-I^64^. On the other hand, while cDC2 induced deletion of OT-I CD8SPs more efficiently, cDC1 contributed more to activation of polyclonal CD8SPs. The basis for this difference is not clear, but one possibility is that cDC2 are more efficient at presenting secreted antigens, such as in RIP-OVA^hi^ thymi, while cDC1 are more efficient at presenting transmembrane antigens expressed by *Aire*^+^ mTEC in the polyclonal setting, consistent with a recent study, showing that CD36 mediates transfer of cell-surface antigens from *Aire*^*+*^ mTEC to cDC1^27^. Alternatively, innate memory CD8^+^ T cells, which comprise 10% of the TCR αβ CD8SP population in C57BL6 thymi and have a strong TCR signaling signature^65^, could interact with cDC1 in the polyclonal setting^66^.

The role of cDC1 versus cDC2 in presenting antigens to CD4SPs is also controversial. Thymic cDC2 has been shown to be more efficient than cDC1 in presenting antigens acquired from circulation on MHC-II, and the appearance of cDC2 in the postnatal thymus coincides with an increased capacity for negative selection^67^. Furthermore, some studies using *Batf3*^−*/*−^ mice, which are deficient for cDC1, found that negative selection^21,29^ and Treg induction^28,29^ remained largely intact. Our imaging studies are consistent with these reports: the total contribution of AIRE^+^ mTECs and cDC2 accounts for approximately two-thirds and three-quarters of the cellular contacts of activated OT-II CD4SP thymocytes in RIP-OVA^hi^ and RIP-mOVA slices, respectively. The cDC2 also interacted three times more frequently than cDC1 with activated polyclonal CD4SPs. In contrast, Perry et al. recently reported that self-antigen transfer from mTEC to cDC1 and presentation on MHC-II plays a significant role in Treg induction^27^. However, while their initial study showed that deletion of 40% of unique CD4SP TCR clones was dependent on bone marrow-derived APCs, they recently found only 2% of the clones required *Batf3*-dependent DCs^27,31^. Thus, it is probable that cDC2 mediates a significant amount of tolerance against TRAs, as we see in our OVA model, with a more specialized role for cDC1 in inducing tolerance to membrane-associated *Aire*-dependent TRAs. Future studies should address this possibility. Given the differential contribution of cDC1 versus cDC2 to thymocyte activation, modulating their frequencies could impact the outcome of central tolerance, consistent with our recent studies in which skewing cDC subtypes alters Treg selection^37^.

The Indo1AM Ca^2+^ indicator dye enables quantification of changes in [Ca^2+^]_i_ in thymocytes in this and previous imaging studies^50–53^. We have limited our analysis to the first 4 h after incubation on thymic slices, because of a sharp increase in calcium-bound dye signal after 6 h, possibly due to the intracellular dye compartmentalization. The development and application of brighter genetically encoded calcium sensors^68,69^ could perhaps shed further light on changes in TCR signaling during negative selection beyond this initial window. It would be interesting to consider whether thymocytes undergoing negative selection versus Treg induction interact with distinct APCs and activate calcium to a different extent in situ. Future studies could also use this approach to examine the contribution of distinct APC subsets to tolerance induction against self-antigens expressed by DCs or other medullary APCs. In conclusion, we have established a 2PM imaging approach to assess the contribution of distinct APC subsets to the induction of central tolerance in a live thymic environment, and have shown that AIRE^+^ mTECs and conventional thymic DC subsets contribute significantly to negative selection of polyclonal and monoclonal CD4SP and CD8SP thymocytes responding to endogenous TRAs.

## Methods

### Mice

C57BL/6J, B6.SJL-Ptprca PepCb (CD45.1), C57BL/6-Tg(TcraTcrb)1100Mjb/J (OT-I), B6.Cg-Tg(TcraTcrb)425Cbn/J (OT-II), B6(Cg)-Rag2tm1.1Cgn/J (*Rag2*^−*/*−^), B6.129S2-Ighmtm1Cgn/J (µMT^−/−^), B6.Cg-Tg(Itgax-Venus)1Mnz/J (CD11c-EYFP)^45^, *Adig*^46^ (Aire-Driven Igrp-Gfp, M. Anderson, University of California San Francisco, San Francisco, USA), *Aire*^EGFP^ ^9^(M. Matsumoto, University of Tokushima, Tokushima, Japan), C57BL/6-Tg(Ins2-TFRC/OVA)296Wehi/WehiJ (RIP-mOVA)^43^, RIP-OVA^hi^ ^44^ (W.R. Heath, University of Melbourne, Melbourne, Australia), and CD11c-mCherry^54^ (M. Krummel, University of California San Francisco, USA) strains were bred in-house. All strains were sourced from Jackson Laboratories, except as specified. Mouse maintenance and experimental procedures were carried out with approval from the Institutional Animal Care and Use Committee at the University of Texas at Austin. Experiments were performed using mice 1–5 months of age of mixed sex. All strains were bred and maintained under specific pathogen-free conditions in the University of Texas at Austin animal facility.

### Antibodies

Antibodies directed against the following mouse markers were used: CD3 (145–2C11, Tonbo Biosciences 60–0031), CD4 (RM4–5, BioLegend 100559; GK1.5, BioXCell BE0003–1), CD8 (53–6.7, Tonbo Biosciences 75–0081; 53.6.72, BioXCell BE0004), CD11b (M1/70, BioLegend 101204; M1/70, BioXCell BE0007), CD11c (N418, BioLegend 117322), CD19 (6D5, BioLegend 115534), CD25 (PC61, BioLegend 102024; PC61.5.3, BioXCell BE0012), CD45 (30-F11, BioLegend 103138), CD45.1 (A20, BioLegend 110714), CD69 (H1.2F3, BioLegend 104504), CD80 (16–10A1, BioLegend 104724), B220 (RA3–6B2, BioLegend 103232; RA3.3A1/6.1, BioXCell BE0067), AIRE (5H12, eBioscience 53–5934–82), EpCAM (G8.8, BioLegend 118206), F4/80 (BM8, BioLegend 123116), Gr-1 (RB6–8C5, BioLegend 108410; RB6–8C5, BioXCell BE0075), I-A/I-E (M5/114.15.2, BioLegend 107628), NK1.1 (PK136, BioLegend 108716), PDCA1 (eBio927, eBioScience 12–3172–82), Sirpα (P84, BioLegend 144008), TER-119 (TER-119, BioLegend 116210; TER-119, BioXCell BE0183), Vα2 (B20.1, BioLegend 127818), Vβ5 (MR9–4, BioLegend 139504), and XCR1 (ZET, BioLegend 148212). For immunostaining ~10^7^ cells in 100 μL of PBS + 2% bovine calf serum (BCS), fluorochrome-conjugated antibodies were diluted from stock concentrations of 0.5 mg mL^−1^ and incubated with cells for 20 min on ice, unless specified.

### Purification of thymocyte subsets

CD8SP cells were enriched from OT-I; *Rag2*^−*/*−^ thymocytes by incubating 2 × 10^8^ cells mL^−1^ with antibodies against CD4 (21 μg mL^−1^) and CD11b, Gr-1, Ter-119, and CD25 (5 μg mL^−1^ each) for 30 min on ice in PBS + 2% BCS, followed by immunomagnetic depletion using sheep anti-rat IgG magnetic DynaBeads (Life Technologies) at a 2:1 cell:bead ratio. Magnetic depletion was repeated with half the number of beads to improve enrichments. CD4SP cells were similarly enriched from OT-II; *Rag2*^−*/*−^ thymocytes using anti-CD8 (96 μg mL^−1^), instead of anti-CD4 with the above antibodies. In polyclonal experiments, CD8SP and CD4SP were isolated from C57BL6/J thymi, as above. Purities of isolated SP thymocytes were determined by flow cytometry (Supplementary Fig. 1a), using the following fluorochrome-conjugated antibodies: CD3, CD4, CD8, and CD69-biotin (all at 1:400), followed by Streptavidin Qdot 605 (Thermo Fisher Scientific, 1:800). Cells were washed and resuspended in 10 µg mL^−1^ propidium iodide (PI) to determine viability. Samples were analyzed on an LSR Fortessa flow cytometer (BD), and data were analyzed using FlowJo (v. 9.8, Tree Star). For each slice, 10^6^ isolated cells were stained with 2 μM CMTPX CellTracker Red and 2 μM Indo1AM (both from Life Technologies) for 30 min at 37 °C in 1.5 mL of the DRPMI medium (RPMI 1640 without L-glutamine, phenol red, and sodium bicarbonate; Cellgro) supplemented with 0.2 g L^−1^ sodium bicarbonate and 20 mM HEPES. Cells were washed and incubated in 1.5 mL of the complete RPMI medium (RPMI 1640 with 2 mM L-glutamine, 50 U mL^−1^ penicillin, 50 mg mL^−1^ streptomycin, and 10% (v/v) fetal bovine serum) for 30 min to destain. Cells were washed twice with complete RPMI medium before incubation on thymic slices^70^.

### Thymic slice preparation

For 2PM imaging of OT-I CD8SP or OT-II CD4SP cells on single-reporter slices, slices were generated from thymi co-expressing either CD11c-EYFP or *Adig*, and RIP-mOVA, RIP-OVA^hi^, or no OVA (WT). For 2PM imaging of polyclonal thymocytes, slices were generated from a CD11c-EYFP or *Aire*^EGFP^ thymus. For dual-reporter 2PM imaging, slices were generated from a CD11c-mCherry^+^ *Aire*^EGFP^ thymus. For negative selection assays, slices were generated from RIP-mOVA, RIP-OVA^hi^, or WT littermate control thymi. Dissected thymi were embedded in 4% (w/v) NuSieve^®^ GTG^®^ low-melting-temperature agarose (Lonza) in PBS at 37 °C. The solidified agarose block was sectioned into 400-µm-thick slices using a VT 1000 S Microtome (Leica) in a bath of ice-cold PBS, with vibratome frequency set to 70 Hz, speed to 0.20 mm s^−1^, and amplitude to 0.6 mm. Slices were collected in DRPMI + 10% BCS on ice before transfer to 0.4 -µm tissue culture inserts (Millipore) in 35-mm Petri dishes containing 1 mL of the complete RPMI medium. For dual-reporter imaging, dishes contained 1 mL of the complete RPMI medium with either 1 nM OVA_257–264_ (New England Peptide) for OT-I stimulation or 100 nM OVA_323–339_ (GenScript) for OT-II stimulation. In total, 10^6^ labeled thymocytes were concentrated into a 20 -µL volume of the complete RPMI medium and carefully pipetted onto the surface of each thymic slice before incubation at 37 °C, 5% CO_2_ to allow migration of thymocytes into the thymic slice.

### Two-photon fluorescence microscopy

After incubation for ≥ 0.5 h, thymic slices were transferred and secured in an imaging chamber (Harvard Apparatus) on the microscope stage. The chamber was perfused with the DRPMI medium supplemented with 2 g L^−1^ sodium bicarbonate, 5 mM HEPES, and 1.25 mM calcium chloride. The perfusion medium was fed by gravity to the stage inlet through a perfusion 300 -mL IV set with the regulator flow rate at ~100 mL h^−1^, or ~1 drop per second. The perfusion medium was aerated with 95% oxygen with 5% carbon dioxide, and maintained at 37 °C with a heated microscope stage and inline perfusion heater. Images were acquired every 15 s, through a depth of 40 µm, at 5 -µm intervals for durations of 15 min, using an Ultima IV microscope with a 20 × water immersion objective (NA 1.0) and PrairieView software (v4.4, Bruker). The sample was illuminated with two MaiTai titanium:sapphire lasers (Newport) tuned to 730 nm (for Indo1AM) and 840 nm (for *Adig* or *Aire*^EGFP^) or 875 nm (for CD11c-EYFP). Emitted light was passed through 400/50, 480/40, 535/50, and 607/45 band-pass filters (Chroma) to separate PMTs for detection of Indo1-high calcium, Indo1-low calcium, EGFP/EYFP, and CMTPX fluorescence, respectively. For dual APC reporter imaging (Supplementary Fig. 2), the sample was illuminated with a MaiTai laser tuned to 730 nm (for Indo1AM) and an InSight laser (Newport) tuned to 900 nm (for *Aire*^EGFP^) and 1040 nm (for CD11c-mCherry). Emitted light was passed through 400/50, 473/24, 525/50, and 605/70 band-pass filters (Chroma) to separate PMTs for detection of Indo1-high calcium, Indo1-low calcium, EGFP, and CMTPX/mCherry fluorescence, respectively. Images were acquired with PrairieView software (v5.4, Bruker).

### Negative selection assays in thymic slices

Thymic slices generated from C57BL/6J, RIP-mOVA, and RIP-OVA^hi^ thymi were incubated for 24 or 48 h at 37 °C/5% CO_2_ on tissue culture inserts in Petri dishes containing 1 mL of the complete RPMI, with or without 10 µM OVA_257–264_ or OVA_323–339_, as positive controls for deletion of OT-I and OT-II thymocytes, respectively. In total, 10^6^ total thymocytes from OT-I or OT-II, and 10^6^ total thymocytes from CD45.1 mice per slice plus one input control were stained in 5 mL of the DRPMI medium supplemented with 0.2 g L^−1^ sodium bicarbonate and 20 mM HEPES, with 5 µM CMF2HC CellTracker Blue (Life Technologies). Cells were washed and resuspended in 5 mL of the complete RPMI medium for 30 min to destain, and then washed twice before application onto each slice. After incubation at 37 °C 5% CO_2_ for the specified times, slices were gently washed twice in PBS and manually disrupted to obtain single-cell suspensions. An aliquot of input thymocytes and slice samples was immunostained for CD3, CD4, CD8, CD69-biotin, CD25, Vα2, Vβ5, and CD45.1, all at (1:400), followed by Streptavidin Qdot 605 (1:800). Samples were resuspended in 10 µg mL^−1^ PI for viability, and 5 × 10^4^ polystyrene beads were added to the tubes to quantify the number of OT-I CD8SP (CMF2HC^+^CD45.1^−^Vα2^+^Vβ5^+^CD8^+^) or OT-II CD4SP (CMF2HC^+^CD45.1^−^Vα2^+^Vβ5^+^CD4^+^) CD25^−^ and CD25^+^ cells both in input samples and within slices after incubation by flow cytometry. OT-I CD8SP or OT-II CD4SP subsets were quantified and normalized for variable slice entry based on the ratio of control CD45.1^+^ CD8SP or CD4SP cells in each slice to the comparable CD45.1^+^ subset in the input sample. To compile data from multiple experiments, cell numbers were normalized to the average cell number of all experiment inputs.

### Flow-cytometric analyses of thymic stroma

CD11c-EYFP, RIP-mOVA, RIP-OVA^hi^, or WT (OVA^−^) thymi were lightly scored with a blade and enzymatically digested in a 2-mL solution of 0.01% (w/v) Liberase + 0.1% DNAase (both from Roche) in PBS for 12 min at 37 °C, gently swirling at 6 min. The supernatant was transferred into 35 mL of PBS + 2% BCS and 5 mM EDTA at 4 °C, and comparable digestion of the remaining tissue fragments was repeated twice to completely dissociate the tissue. The collection volume was brought to 50 mL and filtered through a 70 -µm nylon mesh to achieve a single-cell suspension.

For CD11c-EYFP analysis, cells were immunostained with the following fluorochrome-conjugated antibodies: Ter-119 (1:400), Gr-1 (1:2000), F4/80 (1:200), CD11b (1:200), CD11c (1:400), CD19 (1:200), CD45 (1:300), PDCA1 (1:200), Sirpα (1:200), I-A/I-E (1:400), and XCR1-biotin (1:200), followed by incubation with Streptavidin Qdot 605 (1:800). Samples were washed and resuspended in 10 µg mL^−1^ PI for viability before analysis by flow cytometry (gating shown in Fig. 5a). Cell subsets were quantified based on the following cell markers: cDC1 (Ter-119^−^Gr-1^−^CD11c^+^I-A/I-E^+^CD45^+^Sirpα^−^XCR1^+^), cDC2 (Ter-119^−^Gr-1^−^CD11c^+^I-A/I-E^+^CD45^+^Sirpα^+^XCR1^−^), pDC (Ter-119^−^Gr-1^−^CD45^+^PDCA-1^+^), macrophage (Ter-119^−^Gr-1^−^ CD11b^+^F4/80^+^CD45^+^), and B cell (Ter-119^−^Gr-1^−^CD11c^−^I-A/I-E^+^CD45^+^CD19^+^). For quantification of APCs, cells were immunostained with 1 µg mL^−1^ biotin-conjugated *Ulex europaeus* agglutinin I (UEA-1; Vector Laboratories) and the following fluorochrome-conjugated antibodies: EpCAM (1:800), CD11c (1:400), Sirpα (1:200), XCR1, I-A/I-E (1:400), CD45 (1:300), and Zombie Red (BioLegend, 1:1000) for viability, followed by Streptavidin Qdot 605 (1:800). Cells were then fixed and permeabilized with the Transcription Factor Staining Buffer Kit (Tonbo Biosciences) and stained with an antibody against AIRE (1:200) in a permeabilization buffer. Quantification of cell subsets was conducted by flow cytometry (Supplementary Fig. 5c), based on the following cell markers: AIRE^+^ mTEC (EpCAM^+^CD11c^−^I-A/I-E^+^CD45^−^UEA-1^+^AIRE^+^), cDC1 (EpCAM^−^CD11c^+^I-A/I-E^+^CD45^+^Sirpα^−^XCR1^+^), and cDC2 (EpCAM^−^CD11c^+^I-A/I-E^+^CD45^+^Sirpα^+^XCR1^−^).

### Generation and analysis of bone marrow chimeras

The bone marrow from femurs of OT-II and OT-II; µMT^−/−^ mice was incubated with 25 µg mL^−1^ anti-CD3, followed by magnetic depletion with anti-rat IgG DynaBeads at a 4:1 cell:bead ratio. C57BL6/J and RIP-OVA^hi^ mice were lethally irradiated in two split doses of 450 rad, separated by 3 h, and injected with OT-II or OT-II; µMT^−/−^ bone marrow cells. Thymocyte chimerism was assessed 6 weeks after transplantation by staining with fluorophore-conjugated anti-CD3, CD4, CD8, CD69−biotin, CD25, Vα2, and Vβ5 (all at 1:400), followed by Streptavidin Qdot 605 (1:800). Samples were washed and resuspended in 10 µg mL^−1^ PI for viability before flow-cytometric analysis (Supplementary Fig. 7b).

### FACS purification of thymic stromal cell subsets

RIP-mOVA and RIP-OVA^hi^ thymi were enzymatically digested with Liberase in the presence of DNAase I, as above. For cDNA preparation, cells were immunostained with 1 µg mL^−1^ fluorescein isothiocyanate (FITC)-conjugated UEA-1 and the following fluorochrome-conjugated antibodies: EpCAM (1:800), Ter-119 (1:400), CD11c (1:400), B220 (1:200), I-A/I-E (1:400), CD80 (1:400), and CD45 (1:300). Cells were washed and resuspended with 10 µg mL^−1^ PI for viability before flow cytometry. Cells were sorted based on the following cell markers (Supplementary Fig. 6b): mTEC^lo^ (EpCAM^+^Ter-119^−^CD11c^−^I-A/I-E^lo^CD80^lo^CD45^−^UEA-1^+^), mTEC^hi^ (EpCAM^+^Ter-119^−^CD11c^−^I-A/I-E^hi^CD80^hi^CD45^−^UEA-1^+^), DC (EpCAM^−^Ter-119^−^CD11c^+^I-A/I-E^+^CD45^+^), and B cell (EpCAM^−^Ter-119^−^CD11c^−^I-A/I-E^+^CD45^+^B220^+^).

For use in T-cell activation assays, cells were immunostained with 1 µg mL^−1^ FITC-conjugated UEA-1 and the following fluorochrome-conjugated antibodies: EpCAM (1:800), CD11c (1:400), I-A/I-E (1:400), XCR1 (1:200), Sirpα (1:200), and CD45 (1:300). Cells were washed and resuspended in 10 µg mL^−1^ PI for viability before flow cytometry. Cells were sorted based on the following cell markers (Supplementary Fig. 8a): mTEC^hi^ (EpCAM^+^CD11c^−^I-A/I-E^hi^CD45^−^UEA-1^+^), cDC1 (EpCAM^−^CD11c^+^I-A/I-E^+^CD45^+^ XCR1^+^), and cDC2 (EpCAM^−^CD11c^+^I-A/I-E^+^CD45^+^ Sirpα^+^). Stromal subsets were FACS purified to > 95% purity on a FACSAria II (BDBiosciences).

### cDNA preparation and quantitative PCR

Sorted thymic stromal cells from RIP-mOVA and RIP-OVA^hi^ mice (Supplementary Fig. 6b) were resuspended in TRIzol, RNA was extracted, and cDNA was generated using SuperScript^®^ III First-Strand Synthesis SuperMix (Life Technologies). Real-time PCR experiments were performed on an Applied Biosystems Viia7 instrument using the following primers: OVA forward 5′-GGAGCTTCCATTTGCCAGTG-3′, OVA reverse 5′-CCATCTTCATGCGAGGTAAGTAC-3′.

### T-cell activation assay

Sorted thymic stromal cells from RIP-mOVA and RIP-OVA^hi^ mice (Supplementary Fig. 8a) were combined with 2 × 10^4^ OT-I CD8^+^ T cells, enriched from splenocytes using MojoSort^TM^ Mouse CD8 T Cell Isolation Kit (BioLegend) and labeled with 5 µM carboxyfluorescein succinimidyl ester (CFSE, BDBioscience), at a ratio of 2:1 T cell:APC ratio. As positive and negative controls for T-cell proliferation, WT splenocytes were obtained from C57BL6/J mice and prepared before culture by incubating with or without 50 nM OVA_257–264_ for 30 min before combining at a ratio of 2:1 T cell to APC ratio. The culture was incubated at 37 °C, 5% CO_2_ for 3 days, before staining with the following fluorochrome-conjugated antibodies: CD3, CD8, and Vα2 (all at 1:400). Samples were washed and resuspended in 10 µg mL^−1^ PI for viability, followed by flow-cytometric analysis of CFSE levels of OT-I T cells (Vα2^+^CD3^+^CD8^+^; Supplementary Fig. 8b).

### Imaging analysis

Using Imaris (v8.3, Bitplane), thresholded surfaces were generated manually on thymocytes (CellTracker Red), and stromal cells (EYFP/EGFP) and migratory paths were tracked for thymocytes. For dual-reporter imaging, surfaces were generated on DCs (mCherry) and mTECs (EGFP), as above. Thymocyte surfaces were generated using the Colocalization feature (Imaris, v9.2) to identify CellTracker Red- and Indo1-positive cells. Only thymocytes that were observed for more than 20 time points (≥ 5 min) were included in analyses. Aggregated thymocytes were manually separated in the dataset based on size and morphology. Imaris Statistics was used to generate migration parameters, including mean velocity and straightness.

Indo1 ratios (*R*) were calculated for each thymocyte by dividing the mean cell fluorescence intensity in the Indo1-high calcium channel by that of Indo1-low calcium, for every time point. *R* was then normalized for every imaging experiment by dividing the *R* values by the average Indo1 ratio of all thymocyte time points obtained on the complementary WT controls for that particular experiment (*R*_bl_). For each experiment, the threshold value for high calcium (*R*_th_) was calculated as an increase over *R*_bl_ of 25% of the dynamic range between the highest R value observed in the experiment (*R*_pk_) and *R*_bl_. Thymocytes were considered activated if their average *R* across all time points (*R*_avg_) is greater than *R*_th_. Individual *R* traces were also analyzed to find thymocytes undergoing initial activation, which may be at ~*R*_bl_ at the beginning of acquisition, and thus may not have *R*_avg_ > *R*_th_. These thymocytes were defined as those that have a twofold increase in *R* over 1–2 consecutive time points (within 30 s) and a signal decay half-life greater than four consecutive time points (≥ 1 min).

ImarisXT was used to calculate the distance between every thymocyte and the nearest stromal cell surface for every time point. Activated thymocytes were scored as contacting stroma when the thymocyte was within 3 µm of the stromal cell surface for at least four consecutive time points (≥ 1 min). Contact threshold distance was selected, so that the contacting cells would be within one voxel distance (0.582 × 0.582 × 5-µm voxel size for ×40 magnification), with the 3 -µm threshold determined after manual inspection of datasets. For thymocytes undergoing initial activation, contact must occur within two time points of calcium flux (± 30 s of peak flux) to be scored as contacting stroma during activation. Because of possible false positives detected due to the low signal-to-noise ratio of Indo1 observed on the single-cell level within the tissue, all contacts scored were confirmed by manual inspection of the image set.

Mean contact lifetimes were estimated based on the dataset of activated thymocytes that were in contact with an APC at the first imaging time point of a movie. The fraction of thymocytes remaining in contact with an APC over time was graphed and fit to a first-order exponential decay curve using Prism (v.6, GraphPad), from which the mean lifetime was estimated.

### Statistics

All statistical analysis was performed using Prism, with the corresponding test, and multiple-test corrections were listed in the Figure Legends. Statistical power analysis was conducted using R (v. 3.5.1, R Core Team) in order to ensure a sufficient imaging dataset size to detect differences between conditions with 80% power.

### Reporting summary

Further information on experimental design is available in the Nature Research Reporting Summary linked to this article.

## Supplementary information

Supplementary Information

Reporting Summary

Description of Additional Supplementary Files

Supplementary Movie 1

Supplementary Movie 2

Supplementary Movie 3

Supplementary Movie 4

Supplementary Movie 5

Supplementary Movie 6

Supplementary Movie 7

Supplementary Movie 8

Supplementary Movie 9

Supplementary Movie 10

Source Data

## Data Availability

Source data for Figs. 1f–j; 2; 3b–d; 4b–e; 5b, d, e; and 6c–h; and Supplementary Figs. 1c, 2c 3, 5a–b, 6a, 6c–d, and 7a are provided in the Source Data file. Imaging and other data that support the findings of this study are available from the corresponding author upon reasonable request.
