# Supplementary material for: Live-cell imaging reveals the relative contributions of antigen-presenting cell subsets to thymic central tolerance
Source: Nat Commun. 2019 May 17;10:2220. doi: 10.1038/s41467-019-09727-4 (PMC6525199; doi:10.1038/s41467-019-09727-4)
Supplement: Supplementary file 1 — Supplementary Information [file 41467_2019_9727_MOESM1_ESM.pdf]

Supplementary Information

Live-cell imaging reveals the relative contributions of antigen-presenting cell subsets to thymic central tolerance

J. N. Lancaster, H. M. Thyagarajan, J. Srinivasan, Y. Li, Z. Hu, L. I. R. Ehrlich

Supplementary Figure 1

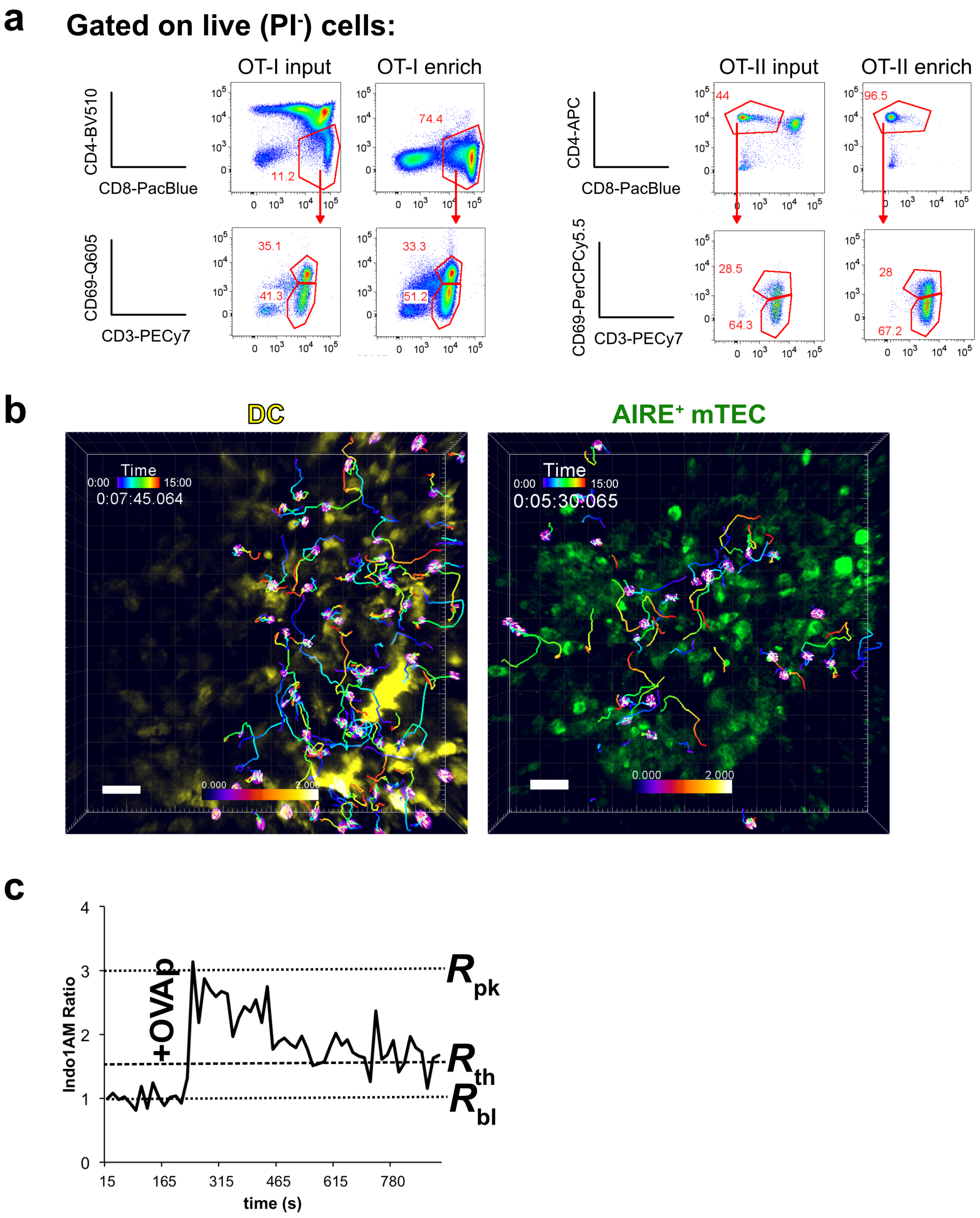

Supplementary Fig. 1, related to Fig. 1. Validation of thymocyte enrichment and determination of threshold for TCR activation from 2PM data of thymocytes undergoing negative selection. (a) Purity of enriched OT-I and OT-II SP thymocytes used in 2PM imaging was determined by flow cytometry. Residual $CD4^-CD8^-$ thymocytes localize to the thymic cortex, and thus are not visualized in 2PM imaging of the thymic medulla throughout. CD69 expression was not upregulated by enriched thymocytes, indicating that the enrichment process did not induce activation artifacts. **(b)** To determine background levels of TCR activation, SP thymocytes were imaged on WT (OVA^-) littermate thymic slices on the same experimental day as they were imaged on OVA^+ thymic slices. Maximum intensity projections of 2PM volumes, at 40X magnification. SP thymocytes are displayed in pseudocolor for $[Ca^{2+}]_i$ ratio on DC (yellow) or $AIRE^+$ mTEC (green) reporter slices. Thymocyte track times are color-encoded as indicated.

Scale bar is 30 µm. See also **Supplementary Movies 3-4**. **(c)** Trace of the average Indo1AM emission ratio of OT-I thymocytes induced to undergo TCR signaling within a WT thymic slice by introduction of exogenous OVA peptide (OVAp). The average baseline $[Ca^{2+}]_i$ (R_{bl}) is indicated prior to addition of OVAp, and the peak $[Ca^{2+}]_i$ (R_{pk}) occurs shortly after OVAp addition. The threshold $[Ca^{2+}]_i$ (R_{th}) above which a cell is considered activated is an increase ≥25% of the range in Indo1AM ratio over baseline: $R_{th} = R_{bl} + 0.25(R_{pk} - R_{bl})$. One calibration experiment shown, with an average of data from 15 thymocytes. Source data are provided as a Source Data file.

Supplementary Figure 2

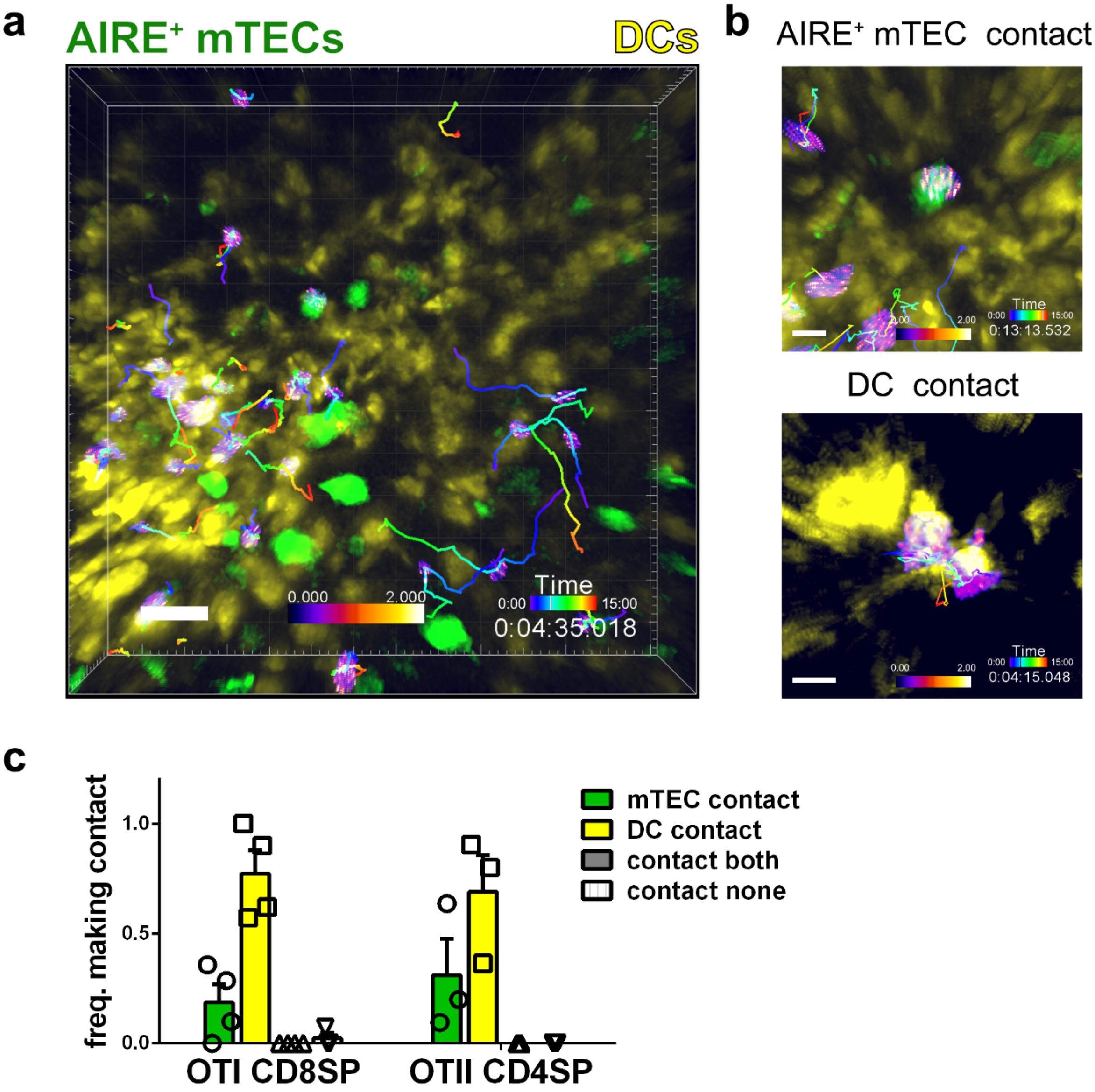

Supplementary Fig. 2, related to Fig. 1. Dual reporter 2PM imaging shows that thymocyte interactions with DCs versus AIRE$^+$ mTECs are discrete events and account for the majority of activation events. (a) To confirm that interactions with AIRE$^+$ mTECs or DCs could be distinguished from one another when both APC subsets were visible, SP thymocytes were imaged on CD11c-mCherry$^+$*Aire*^{EGFP} thymic slices incubated with OVAp. Maximum intensity projections of 2PM volumes are shown, at 40X magnification. SP thymocytes are displayed in pseudocolor for $[Ca^{2+}]_i$ ratio on DC (yellow) and AIRE$^+$ mTEC (green) reporter slices. Thymocyte track times are color-encoded as indicated. Time-lapse imaging through 40 μm depth was carried out with lasers tuned to excite Indo1AM (730 nm), CMTPX and GFP (900 nm), and mCherry (1040 nm). Scale bar is 30 μm. See also **Supplementary Movie 8**. **(b)** Examples of activated OT-II CD4SP (top) and OT-I CD8SP (bottom) thymocytes contacting AIRE$^+$ mTECs or DCs, respectively, on dual reporter thymic slices. Scale bar is 5 μm. **(c)** Frequency with which activated OT-I CD8SPs and OT-II CD4SPs interact with AIRE$^+$ mTECs (green), DCs (yellow), both APCs (gray), or no visible APC (white) on CD11c-mCherry$^+$ *Aire*^{EGFP} thymic slices incubated with OVAp. Data points represent the average of all the cells imaged in a slice, bar shows mean + SEM. Data are compiled from 2 experiments per condition, number of cells: N_{OT-I} = 58, N_{OT-II} = 37. Source data are provided as a Source Data file.

Supplementary Figure 3

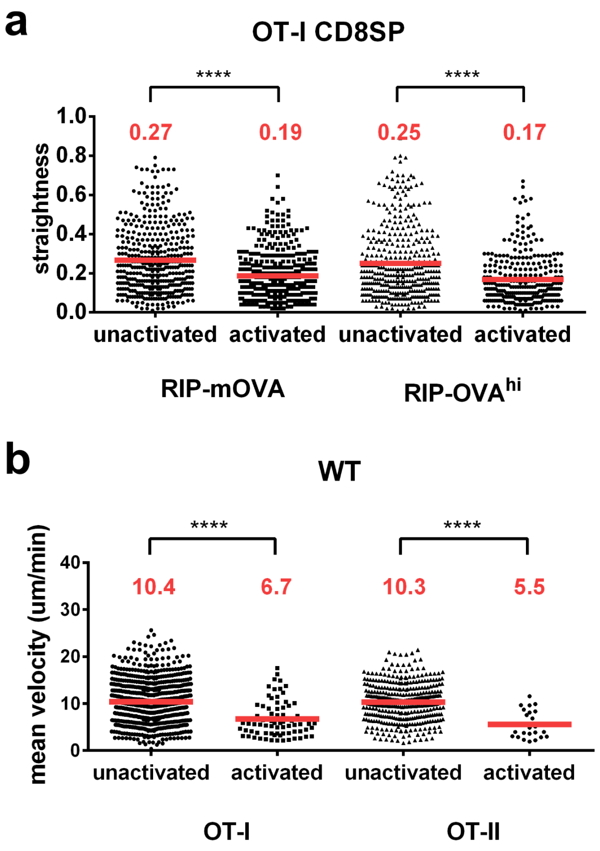

Supplementary Fig. 3, related to Fig. 2. Activated OT-I and OT-II thymocytes migrate with reduced velocities and path straightness. (a) Path straightness of unactivated and activated OT-I CD8SP and OT-II CD4SP thymocytes migrating on RIP-mOVA or RIP-OVA^{hi} thymic slices. Red bar and numbers show mean values. **(b)** Mean cell velocities of unactivated and activated OT-I CD8SP and OT-II CD4SP thymocytes migrating on WT thymic slices. Data points represent individual cells from all experiments and are compiled from the 6-13 experiments per condition analyzed for Figure 1, number of cells: WT ($N_{OT\text{-}I}$ = 827, $N_{OT\text{-}II}$ = 385), RIP-mOVA ($N_{OT\text{-}I}$ = 780, $N_{OT\text{-}II}$ = 1138), RIP-OVA^{hi} ($N_{OT\text{-}I}$ = 738, $N_{OT\text{-}II}$ = 1354). Red bars and numbers show means. Analyzed by unpaired *t*-test, p-value: **** < 0.0001. Source data are provided as a Source Data file.

Supplementary Figure 4

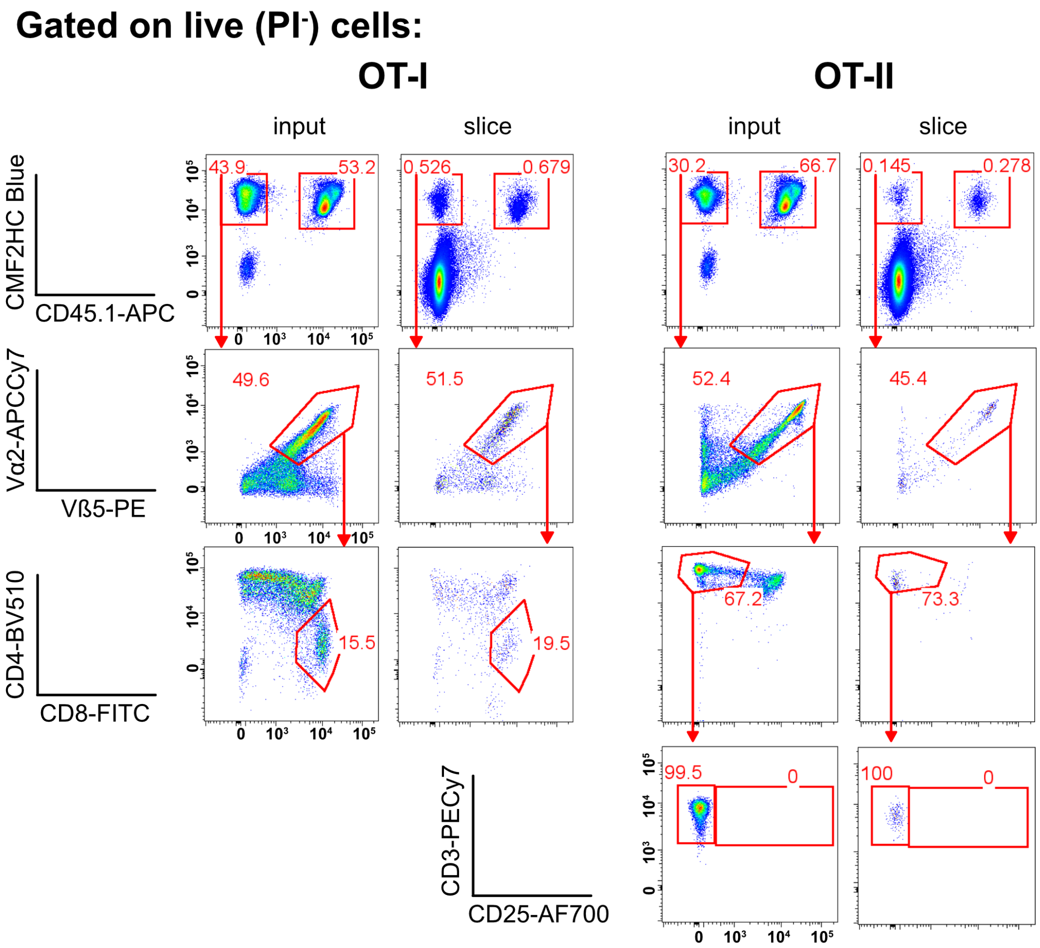

Supplementary Fig. 4, related to Fig. 3. Flow cytometric analysis of OT-I and OT-II thymocytes after incubation in thymic slices. Flow cytometric analysis workflow for quantifying OT-I CD8SP and OT-II CD4SP CD25⁻ and CD4SP CD25⁺ cells remaining 24-48 h after incubation in WT, RIP-mOVA or RIP-OVA^{hi} slices. CD45.1 thymocytes are used to control for differential cellular entry in each slice: OT-I and OT-II cellularity was normalized to the number of CD45.1 thymocytes in each slice. First row is gated on propidium iodide-negative (PI⁻) live, singlet cells. This representative example is from WT slices incubated for 24 hours.

Supplementary Figure 5

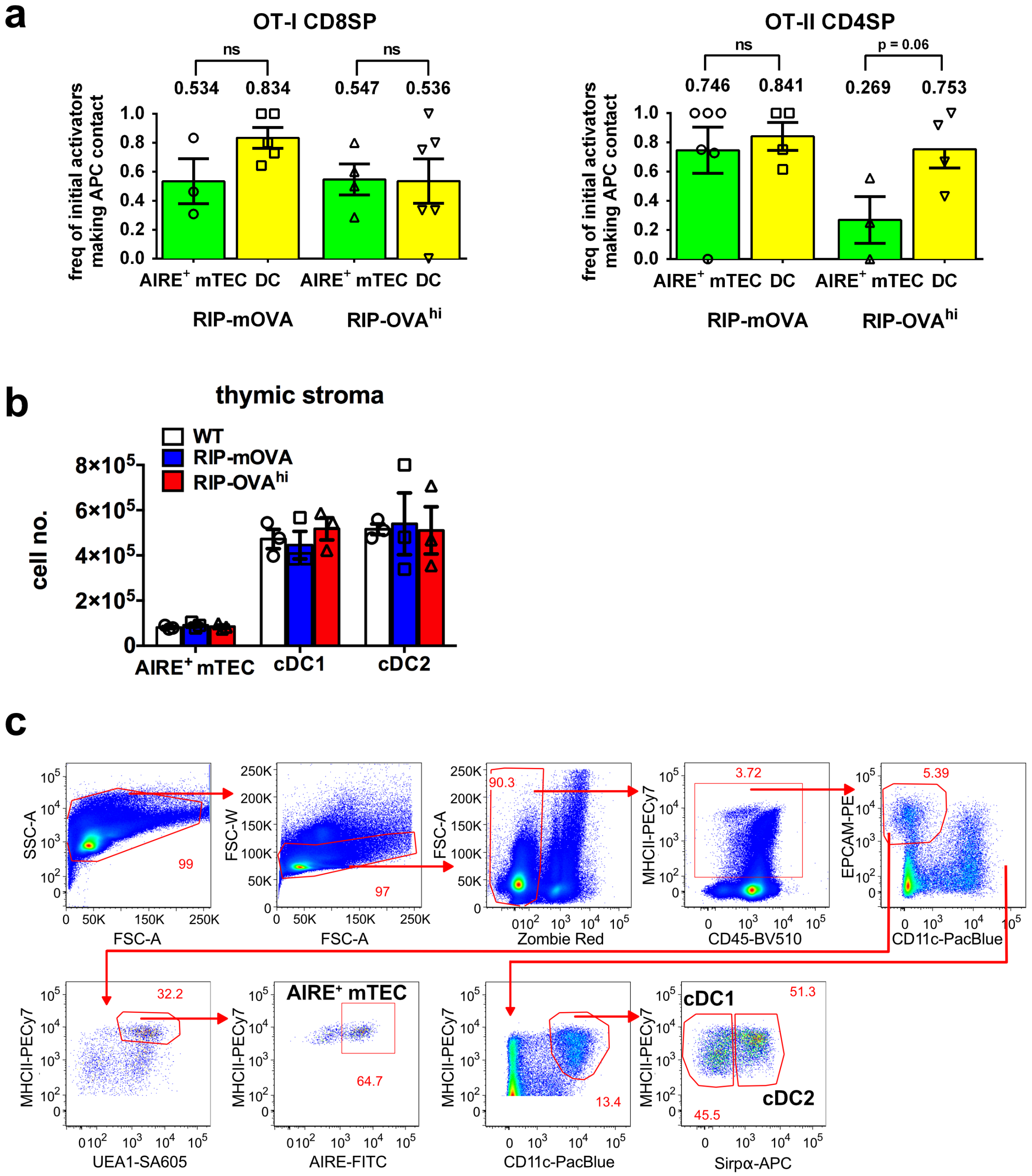

Supplementary Fig. 5, related to Fig. 4. DCs and AIRE$^+$ mTECs constitute the major APC subsets that present OVA TRAs to activate OT-I/II thymocytes. (a) Frequency of OT-I CD8SPs and OT-II CD4SPs undergoing an initial activation event while interacting with AIRE$^+$ mTECs (green) or DCs (yellow) in RIP-mOVA or RIP-OVA^{hi} thymuses. Data points represent the average of all cells in an experiment, and are compiled from the 3-6 experiments per condition analyzed for Figure 4; number of cells: RIP-mOVA ($N_{OT\text{-}I}$ = 60, $N_{OT\text{-}II}$ = 56), RIP-OVA^{hi} ($N_{OT\text{-}I}$ = 38, $N_{OT\text{-}II}$ = 51). Bars represent means ± SEM. Analyzed by unpaired *t*-test, p-value = 0.06, ns: not significant. **(b)** Number of AIRE$^+$ mTECs, cDC1, and cDC2 were quantified by flow cytometry in WT (white), RIP-mOVA (blue), and RIP-OVA^{hi} (red) thymuses. Results are compiled from 2 experiments, with 3 mice total per genotype. Data points represent mice, and bars represent mean ± SEM. Source data are provided as a Source Data file. **(c)** Flow cytometric analysis workflow for quantifying AIRE$^+$ mTECs (MHCII$^+$EPCAM$^+$CD11c$^-$ UEA1$^+$AIRE$^+$), cDC1 (MHCII$^+$EPCAM$^-$CD11c$^+$Sirpα$^-$), and cDC2 (MHCII$^+$EPCAM$^-$CD11c$^+$Sirpα$^+$) in panel b.

Supplementary Figure 6

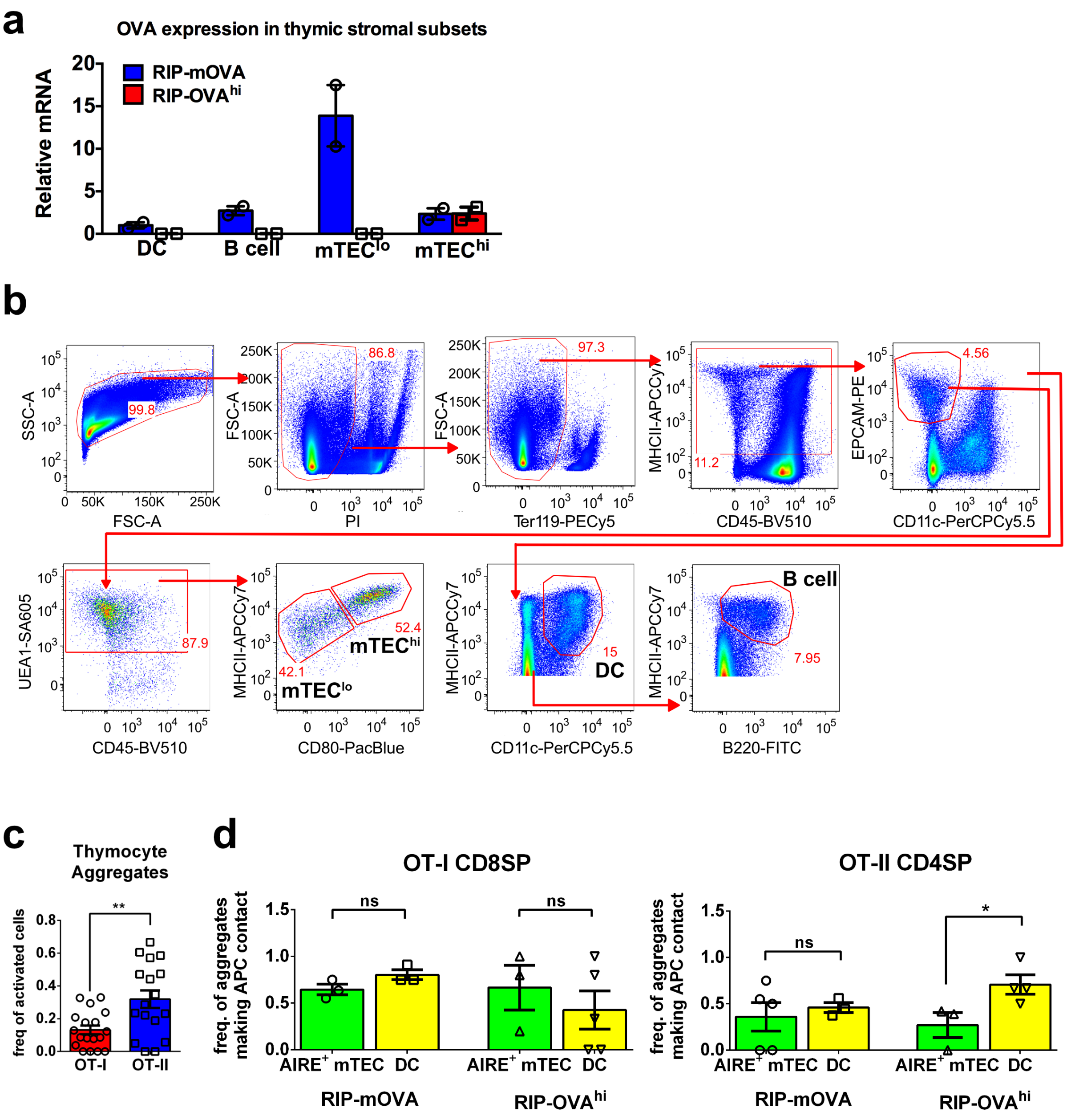

Supplementary Fig. 6, related to Fig. 4. *Ova* is expressed exclusively by mTEC^{hi} cells in RIP-OVA^{hi} mice, and both AIRE^{+} mTEC and DCs can mediate thymocytes clustering. (a) Quantitative RT-PCR expression data for *Ova* transcripts expressed by thymic stromal subsets FACS sorted from RIP-mOVA (blue) and RIP-OVA^{hi} (red) thymuses. Data points represent mean values in individual experiments, and bar represents mean between experiments ± SD. CT values were normalized to β-actin controls within each sample, and then between samples to the RIP-mOVA DC value. Results are compiled from two experiments with three technical replicates each. **(b)** Sorting strategy for isolation of mTEC^{lo} (MHCII^{+}EPCAM^{+}CD11c^{-}UEA1^{+}CD80^{-}), mTEC^{hi} (MHCII^{+}EPCAM^{+}CD11c^{-}UEA1^{+}CD80^{+}), DC (MHCII^{+}EPCAM^{-}CD11c^{+}), and B cells (MHCII^{+}EPCAM^{-}CD11c^{-}B220^{+}) from thymuses for qRT-PCR in panel **a**. **(c)** Frequency of activated OT-I CD8SP (red) and OT-II CD4SP (blue) cells forming aggregates in OVA-expressing thymic slices. **(d)** Frequency of activated OT-I CD8SP and OT-II CD4SP aggregates interacting with AIRE^{+} mTECs (green) or DCs (yellow) in RIP-mOVA or RIP-OVA^{hi} slices. Data points represent the average of all the cells in an experiment, and bars represent means ± SEM. Data are compiled from experiments analyzed in Figure 4. Analyzed by *t*-test, p-value: * < 0.05, ** < 0.01, ns: not significant. Source data are provided as a Source Data file.

Supplementary Figure 7

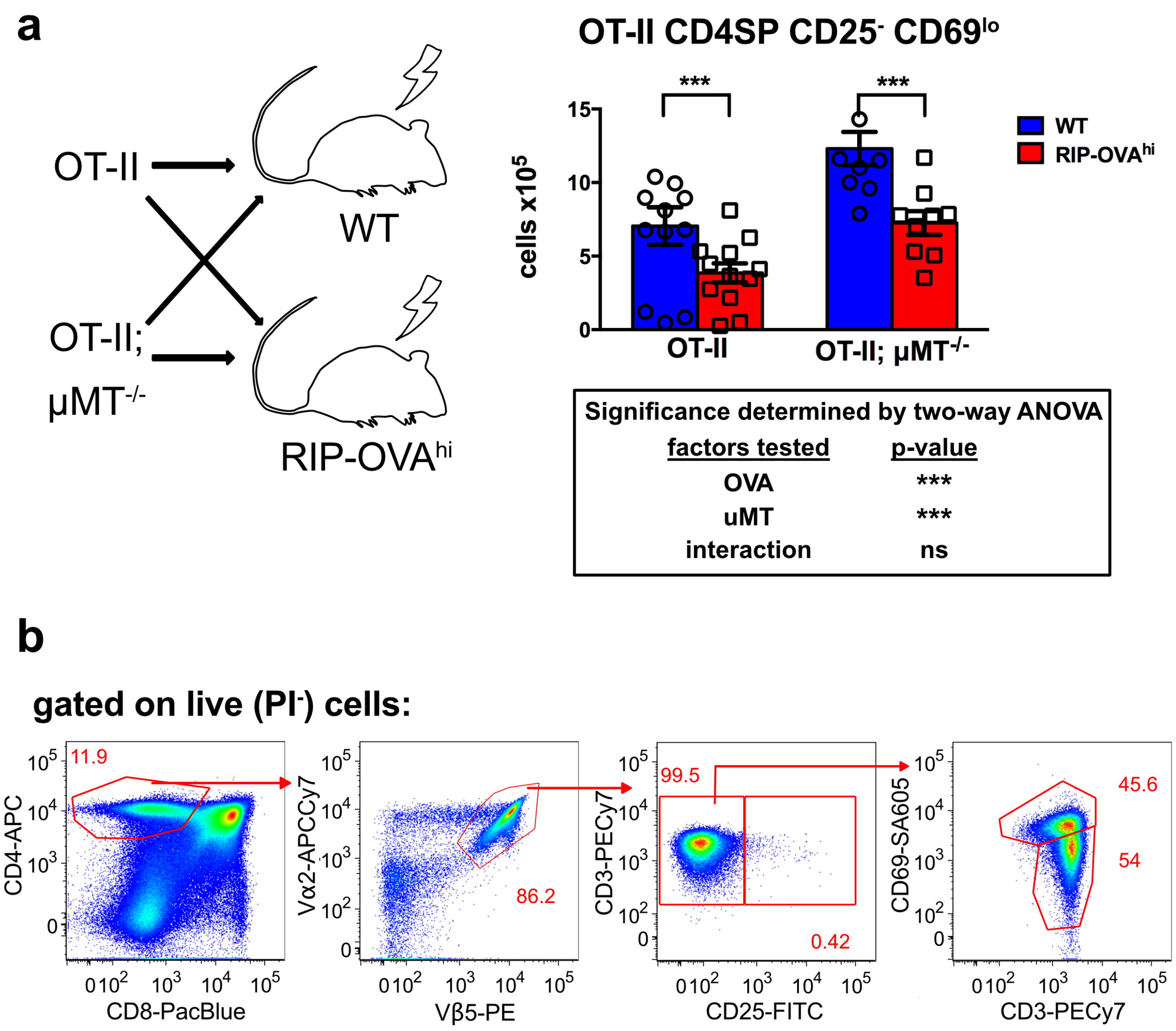

Supplementary Fig. 7, related to Fig. 5. Thymic B cells are not required for OTII deletion against RIP-OVA^{hi}. (a) OT-II (µMT$^{+/+}$) or OT-II; µMT $^{-/-}$ lineage-depleted bone marrow cells were transplanted into lethally-irradiated WT (blue) or RIP-OVA^{hi} (red) recipients, and thymocyte subsets were analyzed by flow cytometry after six weeks. Data points represent individual mice, and bars show the mean ± SEM. Data are compiled from four total experiments, with three mice per group. Analyzed by two-way ANOVA, *** < 0.001, ns: not significant. Source data are provided as a Source Data file. **(b)** Flow cytometric analysis strategy for quantification of OT-II CD4SP (Vα2^{+}Vβ5^{+}CD25^{-}CD69^{lo}) in bone marrow chimeras in **a**.

Supplementary Figure 8

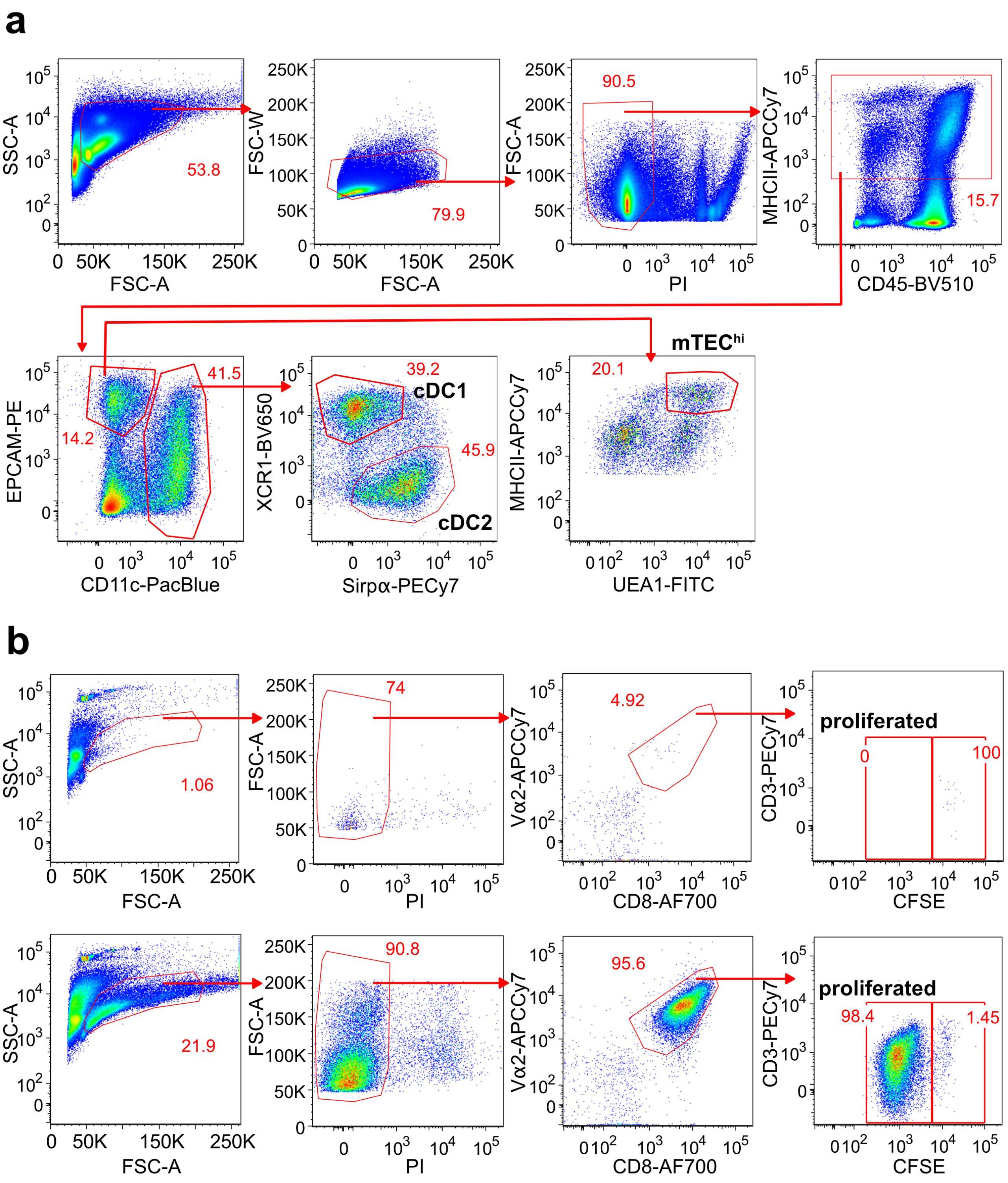

Supplementary Fig. 8, related to Fig. 5. Flow cytometric analysis of OT-I proliferation in response to TRAs presented by sorted thymic APCs. (a) FACS gating strategy for isolation of cDC1, cDC2, and mTEC^{hi} cells from enzymatically-digested RIP-mOVA and RIP-OVA^{hi} thymuses for proliferation assays. **(b)** Gating strategy for flow cytometric analysis of proliferation of CFSE-labeled OT-I CD8^{+} T cells co-cultured with APCs from WT (top) or RIP-mOVA mice (bottom).
