# Supplementary material for: Live-cell imaging reveals the relative contributions of antigen-presenting cell subsets to thymic central tolerance
Source: Nat Commun. 2019 May 17;10:2220. doi: 10.1038/s41467-019-09727-4 (PMC6525199; doi:10.1038/s41467-019-09727-4)
Supplement: Supplementary file 2 — Reporting Summary [file 41467_2019_9727_MOESM2_ESM.pdf]

nature research

Corresponding author(s): Ehrlich, Lauren I. R.

Last updated by author(s): Mar 14, 2019

Reporting Summary

Nature Research wishes to improve the reproducibility of the work that we publish. This form provides structure for consistency and transparency in reporting. For further information on Nature Research policies, see Authors & Referees and the Editorial Policy Checklist.

Statistics

For all statistical analyses, confirm that the following items are present in the figure legend, table legend, main text, or Methods section.

n/a	Confirmed	
☐	☒	The exact sample size (*n*) for each experimental group/condition, given as a discrete number and unit of measurement
☐	☒	A statement on whether measurements were taken from distinct samples or whether the same sample was measured repeatedly
☐	☒	The statistical test(s) used AND whether they are one- or two-sided<br>*Only common tests should be described solely by name; describe more complex techniques in the Methods section.*
☒	☐	A description of all covariates tested
☐	☒	A description of any assumptions or corrections, such as tests of normality and adjustment for multiple comparisons
☐	☒	A full description of the statistical parameters including central tendency (e.g. means) or other basic estimates (e.g. regression coefficient) AND variation (e.g. standard deviation) or associated estimates of uncertainty (e.g. confidence intervals)
☐	☒	For null hypothesis testing, the test statistic (e.g. F, t, r) with confidence intervals, effect sizes, degrees of freedom and P value noted<br>*Give P values as exact values whenever suitable.*
☒	☐	For Bayesian analysis, information on the choice of priors and Markov chain Monte Carlo settings
☒	☐	For hierarchical and complex designs, identification of the appropriate level for tests and full reporting of outcomes
☒	☐	Estimates of effect sizes (e.g. Cohen's *d*, Pearson's *r*), indicating how they were calculated

Our web collection on statistics for biologists contains articles on many of the points above.

Software and code

Policy information about availability of computer code

Data collection	PrairieView (v4.4, Bruker)
Data analysis	Prism (v. 6, GraphPad), Imaris (v8.3, Bitplane), FlowJo (v. 9.8, Tree Star)

For manuscripts utilizing custom algorithms or software that are central to the research but not yet described in published literature, software must be made available to editors/reviewers. We strongly encourage code deposition in a community repository (e.g. GitHub). See the Nature Research guidelines for submitting code & software for further information.

Data

Policy information about availability of data

All manuscripts must include a data availability statement. This statement should provide the following information, where applicable:
- Accession codes, unique identifiers, or web links for publicly available datasets
- A list of figures that have associated raw data
- A description of any restrictions on data availability

Source data for figures are provided with the paper. Imaging and other data that support the findings of this study are available from the corresponding author upon reasonable request.

Field-specific reporting

Please select the one below that is the best fit for your research. If you are not sure, read the appropriate sections before making your selection.

☒ Life sciences ☐ Behavioural & social sciences ☐ Ecological, evolutionary & environmental sciences

Life sciences study design

All studies must disclose on these points even when the disclosure is negative.

Sample size	A mimimum of 3 imaging experiments were conducted per condition (for monoclonal: 2 SP thymocyte subsets x 2 APC reporters x 2 TRAs expressed; for polyclonal: 2 SP thymocyte subsets x 2 APC reporters) with each experiment providing data for >100 thymocytes. The number of experiments was chosen to confirm reproducibility of the experiment and also based on previous literature using 2-photon microscopy, though we included more thymocytes in our analysis.
Data exclusions	Imaging experiments were excluded if thymic slice quality was so poor that cells were unresolved by fluorescence signal, or if enrichment of SP thymocytes was low as determined by flow cytometry.
Replication	At least three independent biologic replicates were carried out for each experiment.
Randomization	Littermate mice were used for antigen positive and negative conditions, which controlled for variations in mouse background and age. When possible, mice were sex-matched, depending on the size of the litter.
Blinding	Imaging data was not analyzed in a blinded fashion, as antigen-negative data were necessary to establish baselines for determining TCR signaling thresholds in antigen-positive data. However, thymocytes within imaging runs were tracked randomly, and independently of their apparent APC contacts or activation status Each track was then quantified in an unbiased fashion to determine if and when cells contacted APCs (<3um distance) and intracellular calcium increased above the threshold.

Reporting for specific materials, systems and methods

We require information from authors about some types of materials, experimental systems and methods used in many studies. Here, indicate whether each material, system or method listed is relevant to your study. If you are not sure if a list item applies to your research, read the appropriate section before selecting a response.

Materials & experimental systems

n/a	Involved in the study	
☐	☒	Antibodies
☒	☐	Eukaryotic cell lines
☒	☐	Palaeontology
☐	☒	Animals and other organisms
☒	☐	Human research participants
☒	☐	Clinical data

Methods

n/a	Involved in the study	
☒	☐	ChIP-seq
☐	☒	Flow cytometry
☒	☐	MRI-based neuroimaging

Antibodies

Antibodies used	Antibodies direct against the following mouse markers were used: CD3 (145-2C11, Tonbo Biosciences), CD4 (RM4-5; GK1.5, BioXCell), CD8 (53-6.7, Tonbo Biosciences; 53.6.72, BioXCell), CD11b (M1/70; M1/70, BioXCell), CD11c (N418), CD19 (6D5), CD25 (PC61; PC61.5.3, BioXCell), CD45 (30-F11), CD45.1 (A20), CD80 (16-10A1), B220 (RA3.3A1/6.1, BioXCell), AIRE (5H12), EpCAM (G8.8), F4/80 (BM8), Gr-1 (RB6-8C5; RB6-8C5, BioXCell), I-A/I-E (M5/114.15.2), NK1.1 (PK136), PDCA (eBio927, eBioScience), Sirpα (P84), TER-119 (TER-119; TER-119, BioXCell), Vα2 (B20.1), Vβ5 (MR9-4), XCR1 (ZET). All antibodies were sourced from BioLegend, unless specified.
Validation	All antibodies used are commercially available, and were validated by the manufacturer. Upon receipt, antibodies were tested in the laboratory using on known positive and negative controls.

Animals and other organisms

Policy information about studies involving animals; ARRIVE guidelines recommended for reporting animal research

Laboratory animals	C57BL6/J mice (Mus musculus) used were 1-5 months of age of mixed sex
Wild animals	N/A
Field-collected samples	N/A
Ethics oversight	The Institutional Animal Care and Use Committee of The University of Texas at Austin approved the mouse maintenance and experiments performed.

Note that full information on the approval of the study protocol must also be provided in the manuscript.

Flow Cytometry

Plots

Confirm that:

☒ The axis labels state the marker and fluorochrome used (e.g. CD4-FITC).

☒ The axis scales are clearly visible. Include numbers along axes only for bottom left plot of group (a 'group' is an analysis of identical markers).

☒ All plots are contour plots with outliers or pseudocolor plots.

☒ A numerical value for number of cells or percentage (with statistics) is provided.

Methodology

Sample preparation	Cells from mouse thymus or thymic slice culture were isolated by manual disruption into buffer or enzymatic digest as noted.
Instrument	BDBiosciences LSR Fortessa, FACSAria II
Software	BDBiosciences FacsDiva for collection, Tree Star FlowJo for analysis
Cell population abundance	Stromal subsets were FACS purified to >95% purity.
Gating strategy	Cells were initially gated based on size FSC/SSC, followed by size FSC to exclude cell doublets. Live cells were then gated based on viability dye before analysis for cellular markers as shown in the Supplement

☒ Tick this box to confirm that a figure exemplifying the gating strategy is provided in the Supplementary Information.
