# Supplementary material for: Live-cell imaging reveals the relative contributions of antigen-presenting cell subsets to thymic central tolerance
Source: Nat Commun. 2019 May 17;10:2220. doi: 10.1038/s41467-019-09727-4 (PMC6525199; doi:10.1038/s41467-019-09727-4)
Supplement: Supplementary file 3 — Description of Additional Supplementary Files [file 41467_2019_9727_MOESM3_ESM.pdf]

Description of Additional Supplementary Files

Supplementary Movie 1, related to Fig. 1. Migration of OT-I CD8SPs among DCs on a RIP-mOVA thymic slice. OT-I CD8SPs (Indo1 ratio) migrate slowly and make contacts with DCs (yellow) while $[Ca^{2+}]_i$ is elevated in most of the imaged cells in the presence of the OVA TRA. Thymocytes are pseudocolored to indicate the Indo1 emission ratio signal, with white to purple denoting high to low $[Ca^{2+}]_i$. Images were acquired for 15 min with 15 sec time intervals, through a depth of 40 µm, and a maximum intensity projection is displayed. Thymocyte paths are color encoded for time; scale bar is 30 µm.

Supplementary Movie 2, related to Fig. 1. Migration of OT-I CD8SPs among AIRE$^+$ mTECs on a RIP-OVA^{hi} thymic slice. OT-I CD8SPs (Indo1 ratio) migrate slowly and make contacts with AIRE$^+$ mTECs (green) while $[Ca^{2+}]_i$ is elevated in most of the imaged cells in the presence of the OVA TRA. Thymocytes are pseudocolored to indicate the Indo1 emission ratio signal, with white to purple denoting high to low $[Ca^{2+}]_i$. Images were acquired for 15 min with 15 sec time intervals, through a depth of 40 µm, and a maximum intensity projection is displayed. Thymocyte paths are color encoded for time; scale bar is 30 µm.

Supplementary Movie 3, related to Supplementary Fig. 1. Migration of OT-I CD8SPs among DCs on a WT thymic slice. OT-I CD8SPs (Indo1 ratio) migrate rapidly among DCs (yellow) while $[Ca^{2+}]_i$ remains near baseline in thymic slices that do not express OVA. The right side of this video is in the medulla, where DCs are present at higher density, while the left half is in the thymic cortex, where DCs are rare. Note that CD8SP thymocytes preferentially migrate within the medulla. This movie was acquired during the same experiment as for **Supplementary Movie 1**. Thymocytes are pseudocolored to indicate the Indo1 emission ratio signal, with white to purple denoting high to low $[Ca^{2+}]_i$. Images were acquired for 15 min with 15 sec time intervals, through a depth of 40 µm, and a maximum intensity projection is displayed. Thymocyte paths are color encoded for time; scale bar is 30 µm.

Supplementary Movie 4, related to Supplementary Fig. 1. Migration of OT-I CD8SPs among AIRE$^+$ mTECs on a WT thymic slice. OT-I CD8SPs (Indo1 ratio) migrate rapidly among AIRE$^+$ mTECs (green) while $[Ca^{2+}]_i$ remains near baseline in thymic slices that do not express OVA. This movie was acquired during the same experiment as for **Supplementary Movie 2**. Thymocytes are pseudocolored to indicate the Indo1 emission ratio signal, with white to purple denoting high to low $[Ca^{2+}]_i$. Images were acquired for 15 min with 15 sec time intervals, through a depth of 40 µm, and a maximum intensity projection is displayed. Thymocyte paths are color encoded for time; scale bar is 30 µm.

Supplementary Movie 5, related to Fig. 1. Initial activation of OT-I CD8SP by AIRE$^+$ mTEC on a RIP-OVA^{hi} thymic slice. An OT-I CD8SP thymocyte (Indo1 ratio) initially migrates rapidly with below threshold $[Ca^{2+}]_i$, then contacts an mTEC (green), slows down and elevates $[Ca^{2+}]_i$ above the activation threshold. Thymocytes are pseudocolored to indicate the Indo1 emission ratio signal, with white to purple denoting high to low $[Ca^{2+}]_i$. Images were acquired for 15 min with 15 sec time intervals, through a depth of 40 µm, and a maximum intensity projection is displayed. Thymocyte paths are color encoded for time; scale bar is 5 µm.

Supplementary Movie 6, related to Fig. 1. Sustained activation of OT-I CD8SP by DCs on a RIP-mOVA thymic slice. An OT-I CD8SP thymocyte (Indo1 ratio) with elevated $[Ca^{2+}]_i$ migrates slowly while contacting a DC (yellow). Thymocytes are pseudocolored to indicate the Indo1 emission ratio signal, with white to purple denoting high to low $[Ca^{2+}]_i$. Images were acquired for 15 min with 15 sec time intervals, through a depth of 40 μm, and a maximum intensity projection is displayed. Thymocyte paths are color encoded for time; scale bar is 5 μm.

Supplementary Movie 7, related to Fig. 1. Sustained activation of an aggregate of OT-II CD4SP cells without contact with a visible APC on a RIP-mOVA slice. OT-II CD4SP thymocytes (Indo1 ratio), with elevated $[Ca^{2+}]_i$ and slow velocities, aggregate and form a ring around a dark region without a visible APC. Nearby DCs (yellow) do not contact the thymocyte aggregate of interest. Thymocytes are pseudocolored to indicate the Indo1 emission ratio signal, with white to purple denoting high to low $[Ca^{2+}]_i$. Images were acquired for 15 min with 15 sec time intervals, through a depth of 40 μm, and a maximum intensity projection is displayed. Thymocyte paths are color encoded for time; scale bar is 5 μm.

Supplementary Movie 8, related to Supplementary Fig. 4. Migration of OT-II CD4SPs among DCs and AIRE$^+$ mTECs on a dual reporter thymic slice. OT-II CD4SPs (Indo1 ratio) migrate slowly after incubation with 100 nM OVAp, and make contacts with AIRE$^+$ mTECs (green) or DCs (yellow) while $[Ca^{2+}]_i$ is elevated in most of the imaged cells. Thymocytes are pseudocolored to indicate the Indo1 emission ratio signal, with white to purple denoting high to low $[Ca^{2+}]_i$. Images were acquired for 15 min with 15 sec time intervals, through a depth of 40 μm, and a maximum intensity projection is displayed. Thymocyte paths are color encoded for time; scale bar is 30 μm.

Supplementary Movie 9, related to Fig. 6. Migration of WT CD4SP cells among AIRE$^+$ mTECs in WT *Aire*^{EGFP} thymic slices. Polyclonal CD4SP thymocytes (Indo1 ratio) migrate rapidly among AIRE$^+$ mTECs (green). Cells were tracked only if present in the imaging field for >5 min. Thymocytes are pseudocolored to indicate the Indo1 emission ratio signal, with white to purple denoting high to low $[Ca^{2+}]_i$. Images were acquired for 15 min with 15 sec time intervals, through a depth of 40 μm, and a maximum intensity projection is displayed. Thymocyte paths are color encoded for time; scale bar is 30 μm.

Supplementary Movie 10, related to Fig. 6. Migration of WT CD8SP cells among DCs in WT CD11c-EYFP thymic slices. Polyclonal CD8SP thymocytes (Indo1 ratio) migrate rapidly among DCs (yellow). Cells were tracked only if present in the imaging field for >5 min. Thymocytes are pseudocolored to indicate the Indo1 emission ratio signal, with white to purple denoting high to low $[Ca^{2+}]_i$. Images were acquired for 15 min with 15 sec time intervals, through a depth of 40 μm, and a maximum intensity projection is displayed. Thymocyte paths are color encoded for time; scale bar is 30 μm.
